# Delineation of metabolic gene clusters in plant genomes by chromatin signatures

**DOI:** 10.1093/nar/gkw100

**Published:** 2016-02-18

**Authors:** Nan Yu, Hans-Wilhelm Nützmann, James T. MacDonald, Ben Moore, Ben Field, Souha Berriri, Martin Trick, Susan J. Rosser, S. Vinod Kumar, Paul S. Freemont, Anne Osbourn

**Affiliations:** 1 John Innes Centre, Norwich Research Park, Norwich, NR4 7UH, UK; 2 Centre for Synthetic Biology and Innovation, Imperial College, South Kensington Campus, London, SW7 2AZ, UK; 3 School of Biological Sciences, University of Edinburgh, King's Building, Edinburgh, EH9 3JR, UK

## Abstract

Plants are a tremendous source of diverse chemicals, including many natural product-derived drugs. It has recently become apparent that the genes for the biosynthesis of numerous different types of plant natural products are organized as metabolic gene clusters, thereby unveiling a highly unusual form of plant genome architecture and offering novel avenues for discovery and exploitation of plant specialized metabolism. Here we show that these clustered pathways are characterized by distinct chromatin signatures of histone 3 lysine trimethylation (H3K27me3) and histone 2 variant H2A.Z, associated with cluster repression and activation, respectively, and represent discrete windows of co-regulation in the genome. We further demonstrate that knowledge of these chromatin signatures along with chromatin mutants can be used to mine genomes for cluster discovery. The roles of H3K27me3 and H2A.Z in repression and activation of single genes in plants are well known. However, our discovery of highly localized operon-like co-regulated regions of chromatin modification is unprecedented in plants. Our findings raise intriguing parallels with groups of physically linked multi-gene complexes in animals and with clustered pathways for specialized metabolism in filamentous fungi.

## INTRODUCTION

The plant kingdom is well known for its great capacity to synthesize diverse specialized metabolites. These natural products have important ecological functions, providing protection against biotic and abiotic stresses such as pest and pathogen attack, ultraviolet radiation and drought. They also provide a rich source of high-value compounds such as agrochemicals and pharmaceuticals, including around 25% of natural product-derived drugs. The ability to produce particular types of natural products is often restricted to narrow taxonomic groupings and is therefore likely to be a reflection of adaptation to different environmental niches.

It has recently become apparent that the genes for the biosynthetic pathways for numerous different types of specialized metabolites are organized in clusters in plant genomes (1–3). In eukaryotes genes for multi-step processes are normally dispersed throughout the genome, except for clusters of tandemly duplicated genes [e.g. *β*-*globin, Hox* loci (animals), and disease-resistance genes (plants)]. However, clusters of functionally-related non-homologous genes are known, including the major histocompatibility complex (MHC) locus in animals and specialized metabolic pathways in fungi. Metabolic gene clusters in plants typically consist of three to ten or more co-localized genes encoding different types of biosynthetic enzymes required for the synthesis of a particular compound or group of compounds. They range in size from 35 kb to several hundred kb (1,3). Examples include clusters for the synthesis of agronomically and pharmaceutically important natural products such as anti-tumour alkaloids from opium poppy (noscapine), anti-nutritional steroidal alkaloids from tomato and potato (α-tomatine, α-solanine/α-chaconine) and triterpenes associated with bitterness in cucumber (cucurbitacenes) (4–6). Cluster-derived plant natural products also have key roles as defence compounds in both monocots and eudicots, for example the cyclic hydroxamic acids 2,4-dihydroxy-1, 4-benzoxazin-3-one (DIBOA) and 2,4-dihydroxy-7-methoxy-1,4-benzoxazin-3-one (DIMBOA) in maize; triterpene glycosides known as avenacins in oat; momilactone, phytocassane and oryzalide diterpenes in rice; and cyanogenic glycosides in sorghum, *Lotus japonicus* and cassava (7–11).

The genes for some of the best known plant natural product pathways such as those for flavonoids and glucosinolates are not clustered. It is not clear why some pathways are clustered and others are not. Intriguingly, clustered plant metabolic pathways have not arisen by horizontal gene transfer from microbes, but have formed relatively recently in evolutionary time by recruitment and neofunctionalization of genes from elsewhere in the genome to establish co-adapted gene complexes (12,13). The mechanisms of cluster formation are not yet understood. However, clustering presumably reflects extreme selection for the assembly of co-adapted alleles of pathway genes. The genes in these metabolic gene clusters are tightly regulated and are expressed only in particular cell types, at certain stages of development, and/or in response to specific environmental triggers (3). Very little is currently known about how these pathways come to be co-ordinately expressed. So far only two transcription factors have been identified, one implicated in regulation of the cucurbitacin cluster in cucumber and the other in indirect regulation of the rice momilactone and phytocassane/oryzalide diterpene clusters (6,14). Physical clustering has the potential to enable fine tuning of cluster expression, since localized chromatin modifications can influence access of transcription factors to pathway genes (15). This fine tuning may be important in ensuring that newly evolved biosynthetic pathways with potentially maladapted intermediate phenotypes are kept under strict control.

Here, we show that metabolic gene clusters in the model plant species *Arabidopsis thaliana* are delineated by blocks of two different types of chromatin marks, histone H3 lysine 27 trimethylation (associated with cluster repression) and histone variant H2A.Z (associated with cluster activation) and that these features can be exploited in genome-wide mining approaches for cluster discovery. We further show that cluster-specific chromatin modifications mark metabolic gene clusters not only in *A. thaliana* but also in oat and maize. Our work opens up new avenues for investigations of specialized metabolism and genome architecture in plants.

## MATERIALS AND METHODS

### Plant material and growth conditions

All *A*.*thaliana* plants used in this study were of the Columbia-0 (Col-0) accession. *pkl pkr2, clf29* were kindly provided by Claudia Köhler (16) and *clf28* (SALK_139371) by Justin Goodrich (17). For analysing the role of SWR1 complex and H2A.Z we used previously described *arp6–1* (18), *pie1–2* (19), *swc6–1* (20) and *hta9–1 hta11–1* alleles (21). Oat experiments were performed with *Avena strigosa* accession S75 (22). Seeds of *A. thaliana* wild-type and mutant lines were surface-sterilized and grown on ¼ Murashige and Skoog medium at 22°C with a 16 h light/8 h dark photoperiod. Oat seeds were surface sterilized and grown on wet filter paper at 22°C with a 16 h light/8 h dark photoperiod.

### Transcript analysis

Total RNA was extracted with the RNeasy Plant Mini Kit (Qiagen) and cDNA was synthesized using the SuperScript III (Invitrogen) reverse transcriptase with oligo-d(T) primers. qPCR was performed with the Sigma-SYBR^®^ Green JumpStart™ Taq ReadyMix™ (Sigma) on a CFX96 Real-Time polymerase chain reaction (PCR) detection system (Bio-Rad). The following cycling conditions were applied: 95°C for 2 min, followed by 40 cycles of DNA denaturation (96°C for 10 s), primer annealing (60°C for 10 s) and extension (72°C for 15 s) and a final elongation step at 72°C for 2 min. The *A. thaliana Actin2* gene was used as a reference gene for quantification. qRT-PCR primers are shown in Supplementary Table S10.

### RNA-seq analysis

RNA-seq data generated from wild-type, *arp6, pie1, swc6* and *hta9 hta11* double mutants was analysed to study the effect of SWR1c and H2A.Z on cluster gene regulation. In brief, total RNA from 14-day-old seedlings grown at 22°C were used for RNA-seq analysis on Illumina HiSeq 2500 using 50-bp single-end sequencing. TopHat v2.0.8 (23) was used to align RNA-seq reads to the *A. thaliana* TAIR10 reference genome assembly. Differential gene expression analysis was carried out using Cuffdiff v2.0.2 (24) on reads per kilobase per million mapped reads (RPKM) values generated using Strand NGS (Agilent). All analysis parameters used were appropriate for single-end reads. Significance of expression change was determined based on the *P*-value corrected for multiple hypothesis testing and a false discovery rate of 0.05. Transcriptional mis-regulation relative to the wild-type was calculated using the RPKM values. The differential expression output file was analysed to find contiguous stretches of three or more genes with similar mis-regulation (at least 2-fold up- or downregulated) across at least three of the four mutants.

### ChIP analysis

ChIP assays were carried out as described in Song *et al*. (25). Trimethyl-histone H3 lysine 27 was assayed using anti-trimethyl-histone H3 lysine 27 from Millipore/Upstate (catalogue no. 07–449). Histone H3 levels were assayed using anti-H3 core antibody from Abcam (catalogue no. 1791). After immunoprecipitation, DNA was recovered using Chelex 100 resin (Bio-Rad, 10 g per 100 ml ddH2O). All ChIP experiments were quantified by quantitative PCR (qPCR) in triplicate with appropriate primers (Supplementary Table S10). Data are represented as the ratio of H3K27me3-precipitated DNA at locus of interest/H3-precipitated DNA at locus of interest.

### Genome mining for H3K27me3-marked co-expressed genes

Initially we analysed data from a total of 1204 *A. thaliana* microarray experiments to identify groups of physically linked (within 10 genes) co-expressed genes using a maximal clique graph-based method. Microarray expression data (Affymetrix GeneChip Arabidopsis ATH1–121501 microarrays) were obtained from AtGenExpress and NASC (Supplementary Data Set 7). Normalization of data was performed using the R package Affy (26), part of the Bio-Conductor project (27). Background correction was done using the robust multi-array average method with quantile normalization. Finally median polishing was applied, leaving log_2_ expression measures. The genefilter R package was used to remove probe sets that had no equivalent TAIR10 mapping, and also to eliminate redundant probe-sets which map to the same transcript, with those reporting the largest interquartile range in raw expression values kept. The plastid and mitochondria genomes were also excluded.

A Pearson correlation coefficient (PCC) matrix was calculated from 1204 microarrays. From the correlation matrix, an off-diagonal slice of width *g* + 1 together with a PCC cutoff, c, was used to construct an undirected, unweighted graph. Edges between nodes (genes) defined as duplicates were removed. Pairs of duplicate genes were defined as coding sequences with BLASTn *e*-values < 0.2. In order to find co-expressed gene clusters, two parameters were defined: the maximum separation between two co-expressed genes, *g*, and the minimum PCC cutoff, *c*. The values of *g* (10) and *c* (0.65) were found by maximizing the average clustering coefficient (ACC) which is a measure of graph complexity. Putative gene clusters were then found by two different methods. In the first most stringent method, maximal cliques with a minimum size of three nodes were found. If two maximal cliques were found to share common nodes these were then grouped together into one cluster. This method resulted in 197 putative gene clusters (Supplementary Data Set 1, Supplementary Script). In a second less stringent method (the subgraph method) clusters were found by searching for connected components of the graph of a minimum size of three nodes. In this case each node in a cluster is only required to be connected one other node in the cluster. This less stringent approach resulted in 452 clusters (Supplementary Data Set 3). Both methods produced significantly more clusters than expected by randomly shuffling the gene order to produce artificial chromosomes (Figure 4B and Supplementary Figure S3).

We next searched the available published data on genome-wide analysis of H3K27me3 modifications in *A. thaliana* (28) to identify regions containing four or more adjacent H3K27me3-marked genes. One hundred sixty-two clusters of four or more adjacent H3K27me3 marked protein coding genes were found in the *A. thaliana* genome compared to a mean of 19 clusters in randomly shuffled artificial chromosomes (Figure 4C, Supplementary Data Set 2). We then compared this list of 162 H3K27me3 marked clusters with the inventory of 197 co-expressed clusters and identified co-expressed regions that contained a minimum of four contiguous H3K27me3-marked genes of which at least three were co-expressed. We chose the clique co-expression regions for comparison to increase the stringency in our analysis. Regions consisting of tandem arrays only of one or two different gene types were then eliminated (BLASTn *e*-values < 0.01), to give a final list of seven putative clusters that contained genes encoding a minimum of three different types of predicted product (Supplementary Table S4).

Analysis of stretches of three contiguous H3K27me3-marked genes and comparison to the co-expression regions did not result in the identification of additional overlapping clusters with the above mentioned criteria.

An identical co-expression cluster analysis was carried out on 619 Affymetrix GeneChip Maize Genome (GPL4032) microarrays downloaded from the Gene Expression Expression Omnibus (GEO) repository (Supplementary Data Set 8). The latest available Maize annotation file (version 35) was downloaded from the Affymetrix website (http://www.affymetrix.com/support/technical/annotationfilesmain.affx) to map probesets to transcripts. The probesets with the largest interquartile range were retained. This analysis resulted in 30 high stringency and 83 low stringency clusters (Supplementary Data Sets 4 and 5). The maize microarray covered around 25% of annotated protein coding sequences compared to the *Arabidopsis* microarray covering 80% of annotated protein coding sequences. This sparse coverage explained the lower number of discovered clusters. However, random shuffling showed statistical significance for these clusters (Supplementary Figure S4).

## RESULTS

### Metabolic gene clusters in *A. thaliana* are characterized by their H3K27me3 silencing marks

**Figure 1.**
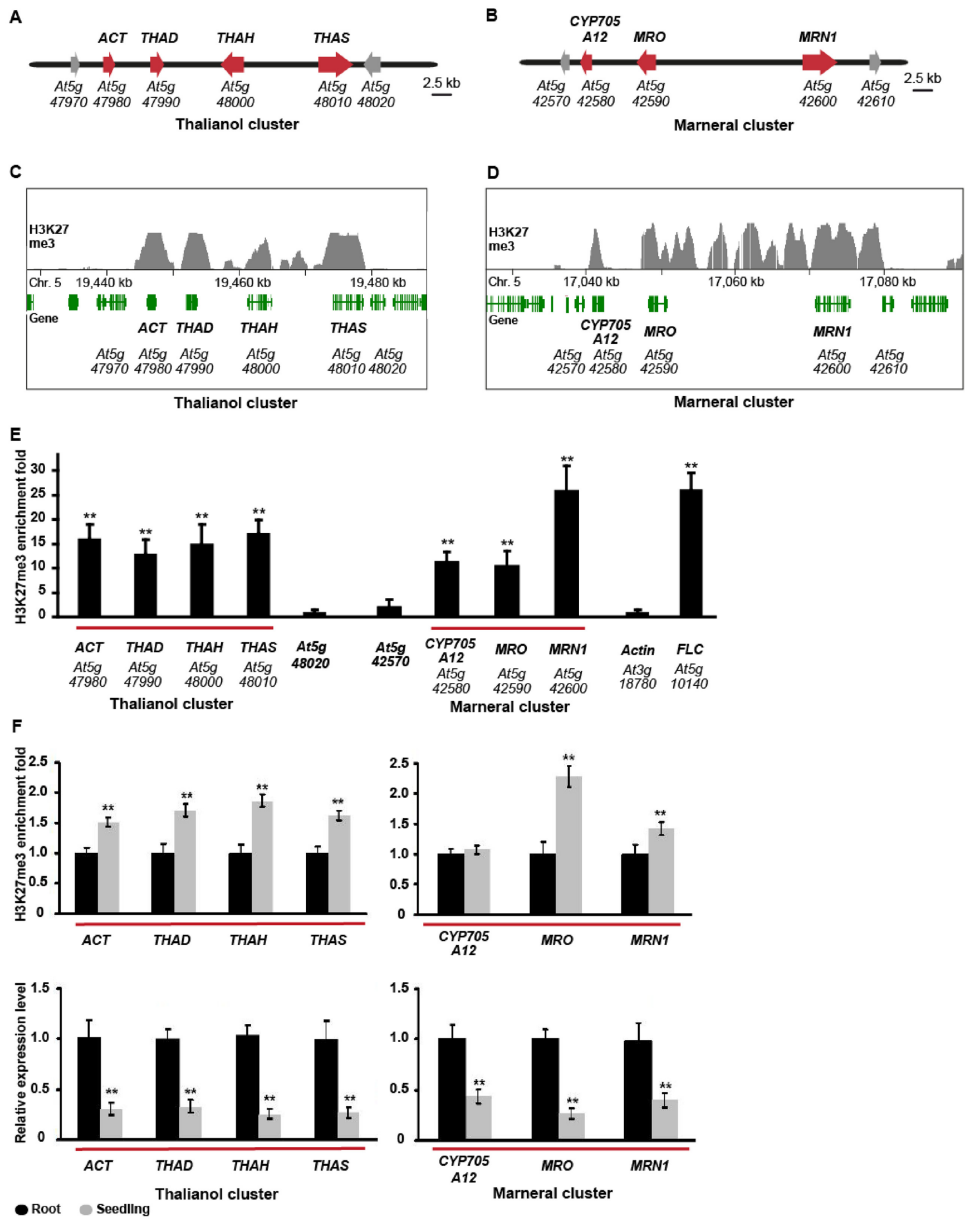
Analysis of H3K27me3 marking and gene expression at the thalianol and marneral clusters. (**A** and **B**) The thalianol and marneral clusters. Red, cluster genes; grey, flanking genes. (**C** and **D**) H3K27me3 ChIP-on-chip data for the two clusters [data extracted from Zhang *et al*. (28)]. The dataset (GSE7064) was uploaded with the University of California, Santa Cruz (UCSC) Genome Browser (University of California, Los Angeles installation). Genes are indicated in green and ChIP-on-chip marks in grey. (**E**) H3K27me3 ChIP analysis of the thalianol and marneral clusters. Six day-old seedlings of *Arabidopsis thaliana* Col-0 were used. Controls were flanking genes, *Actin* and *FLC* (positive control). Cluster genes are underlined in red. **P (*t*-test) < 0.01 for *FLC* and cluster genes compared to flanking gene. (**F**) H3K27me3 ChIP analysis (top) and transcript levels (bottom) for the thalianol and marneral gene clusters in whole seedlings (grey bars) and roots (black bars). The error bars for the ChIP analysis indicate standard deviation for three biological replicates. Transcript levels were analysed by qPCR with *Actin2* as the reference gene; data are shown as means ± sd of three biological replicates. **P < 0.01.

Previously, we identified and characterized two metabolic gene clusters from the eudicot *A. thaliana*, for the synthesis and modification of the triterpenes thalianol and marneral, respectively (Figure 1A and B; Supplementary Figure S1) (12,29). These two clusters have evolved independently and are expressed in different cell types in roots (12,29). Our examination of data from a genome-wide analysis of histone modifications in *A. thaliana* carried out by the Jacobsen lab (28) suggested that the thalianol and marneral clusters are pronouncedly marked by H3K27me3 (Figure 1C and D). These marks are found in both the bodies of the genes and the intergenic regions and distinguish the cluster from the surrounding DNA regions, which do not have pronounced H3K27me3 modifications. Using chromatin immunoprecipitation (ChIP) analysis we confirmed that the genes within these clusters have high levels of H3K27me3 relative to the immediate flanking genes (e.g. *At5g48020* and *At5g42570*) and the actin gene, which was included as a negative control (Figure 1E). *FLOWERING LOCUS C* (*FLC*), which is negatively regulated by H3K27me3, was included as a positive control (30). H3K27me3 is a well-known chromatin mark that is associated with transcriptional repression throughout the eukaryotes. In plants, it is well known to be important for regulation of developmental genes (31). Most H3K27me3 target genes are expressed at low levels, usually in a very tissue-specific manner (28,31). ChIP analysis showed that overall H3K27me3 accumulation at the thalianol and marneral cluster genes is lower in the roots (where these pathways are active) compared to whole seedlings, and that H3K27me3 accumulation is inversely correlated with cluster gene transcript levels, consistent with a potential role for H3K27me3 in cluster repression (Figure 1F).

**Figure 2.**
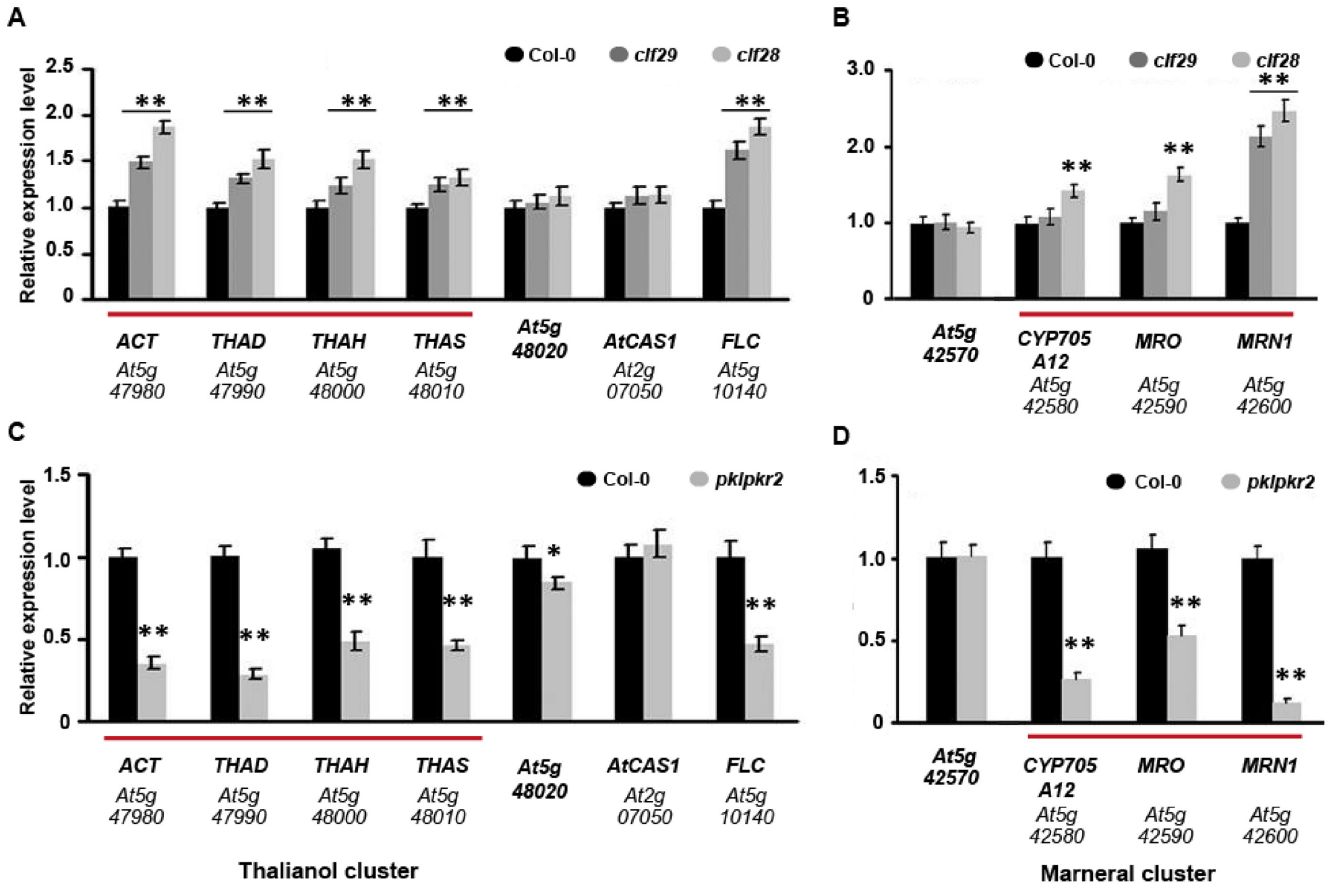
Expression of the *A. thaliana* thalianol and marneral gene clusters is negatively regulated by PcG proteins and positively regulated by PKL/PKR2. (**A** and **B**) qRT-PCR analysis of the transcript levels of the *A. thaliana* thalianol and marneral cluster genes in roots of the wild**-**type Col-0 line and the CLF loss-of-function lines *clf28* and *clf29*. (**C** and **D**) qRT-PCR analysis of the transcript levels of the *A. thaliana* thalianol and marneral cluster genes in roots of the wild**-**type Col-0 line and the *pkl/pkr2* double mutant. Cluster genes are underlined in red. Controls include genes immediately flanking the clusters (gene IDs in bold), the cycloartenol synthase gene *AtCAS1* (which is expressed through different plant tissues) and *FLC* (a known target for H3K27me3 (30) and positive regulation by PKL and PKR2 (16)). Data are means of three biological replicates ± sd; **P (*t*-test) < 0.01 compared with the wild-type.

We next analysed mutant lines of known negative (CURLY LEAF, CLF) and positive regulators (PICKLE, PKL) of H3K27me3 marked genes in *A. thaliana*. CLF is a subunit of the Polycomb Repressive Complex 2 (PRC2) that catalyses trimethylation of H3K27 (17,28,32). We found increased expression of the cluster genes in two independent CLF loss-of-function mutants (Figure 2A and B) and confirmed reduced H3K27me3 levels in these mutants (Supplementary Figure S2A and B). PKL and its homolog PICKLE RELATED 2 (PKR2) have been shown to have trithorax group protein-like functions in *A. thaliana* and to counteract H3K27me3 mediated gene silencing (16,33–34). Consistent with the positive regulatory role of PKL, the transcript levels of the thalianol and marneral cluster genes were significantly reduced in the *pkl pkr2* double mutant (Figure 2C and D). Of note, in a previous genome-wide investigation of PKL function in *A. thaliana* the thalianol cluster gene *ACT* was identified as one of the direct targets of PKL (16). Both CLF- and PKL-dependent changes in mRNA levels were restricted to the gene clusters and did not extend to the immediately adjacent genes.

In animal genomes H3K27me3 marks cover large chromosomal domains and are involved in the co-ordinate expression of co-localized genes within these domains (35–37). In contrast, plant genomes have been reported to lack such domains of H3K27me3 and genome-wide correlations between the expression patterns of neighbouring genes covered by the same H3K27me3 regions are similar to those for randomly paired H3K27me3-marked genes (28). The clustered metabolic pathway genes under investigation here are marked by areas of dense H3K27me3 and show concerted expression. Thus, they represent exceptions to the general H3K27me3 domain structure in plants.

### Association of H3K27me3 with metabolic gene clusters in oat and maize

**Figure 3.**
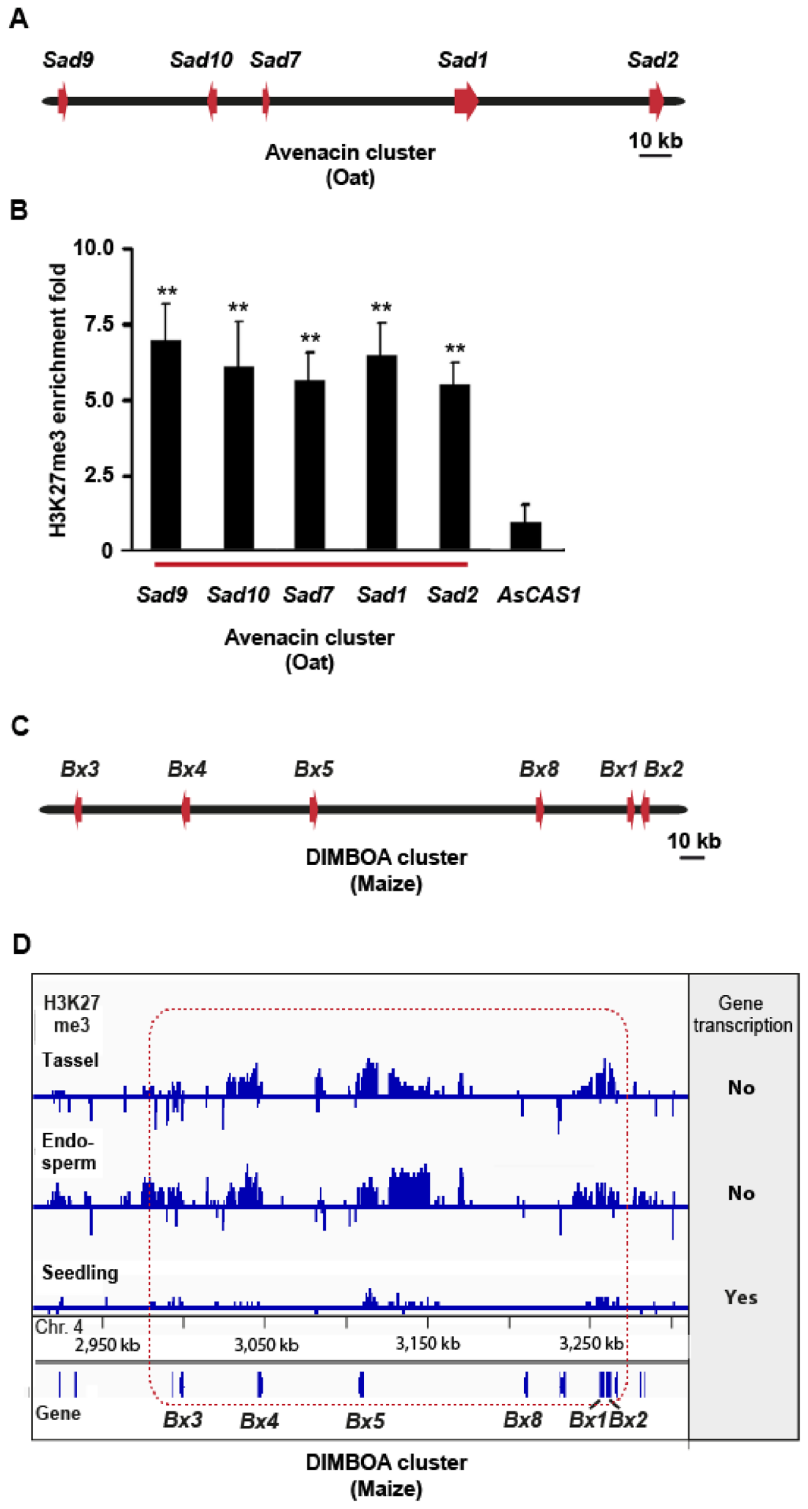
Analysis of H3K27me3 markings at the oat avenacin cluster and the maize DIMBOA cluster. (**A**) The oat avenacin biosynthetic gene cluster. (**B**) H3K27me3 ChIP analysis of avenacin cluster genes. Roots of wild-type *Avena strigosa* seedlings (3-days old) were used. The *A. strigosa* cycloartenol synthase gene *AsCAS1*, which is unlinked to the cluster and is expressed throughout the leaves and roots, was included as a control. Error bars indicate standard deviation of three biological replicates. **P (*t*-test) < 0.01 for cluster genes compared to *AsCAS1*. (**C**) The *Zea mays* DIMBOA cluster. (**D**) ChIP-on-chip data showing H3K27me3 marking at the DIMBOA cluster [data extracted from Makarevitch *et al*. (39)]. The dataset (GSE39456) was uploaded with the Integrative Genomics Viewer (Broad Institute). Gene transcription states of the cluster as extracted from Makarevitch *et al*. (39) are indicated on the right. The DIMBOA gene cluster is framed in red.

Next, we analysed H3K27me3 markings at two metabolic gene clusters in monocots that exhibit distinct expression patterns and encode biosynthetic genes for different types of natural products. The genes for the biosynthesis of the antimicrobial defence compound avenacin, a saponin, are clustered in the genome of oat (8,38) (Figure 3A; Supplementary Figure S1). The avenacin cluster shows a highly restricted expression pattern and is active only in the epidermal cell layer of the root meristem (38). ChIP analysis indicates that the genes in this cluster have strong H3K27me3 markings (Figure 3B). The gene for the first step in the avenacin pathway (*Sad1*) has been recruited from *AsCAS1*, a gene required for the synthesis of essential sterols, by gene duplication, relocation and neofunctionalization (8). *AsCAS1* does not show the strong H3K27me3 marking observed for the avenacin biosynthetic genes (Figure 3B). The first metabolic gene cluster described in plants was the DIMBOA cluster in maize (7). It contains the biosynthesis genes for the formation of hydroxamic acid defence compounds (Figure 3C; Supplementary Figure S1). Analysis of available genome-wide H3K27me3 profiles for maize (39) revealed that the genes in this cluster are also marked with H3K27me3 (Figure 3D). As for the thalianol and marneral clusters, the whole cluster showed a dense yet interrupted H3K27me3 profile with H3K27me3 marks associated with the genes and also in parts of the intergenic regions. The extent of H3K27me3 marking of each of the DIMBOA cluster pathway genes *Bx1, Bx3,Bx4* and *Bx5* was inversely correlated with cluster expression, similar to our finding for the *A. thaliana* clusters (Figure 3D; Supplementary Table S1).

Collectively our results indicate that clusters of plant natural product genes have strong H3K27me3 markings, and suggest that H3K27me3 may be involved in restricting cluster expression in both *A. thaliana* and monocots. We note that genes that are dispersed around plant genomes can still be co-ordinately regulated and may also be subject to Polycomb repression. To investigate the possibility that pathway genes for plant specialized metabolism may be generally marked by H3K27me3 irrespective of whether they are co-localized or dispersed in the genome we analysed the association of this chromatin mark with non-clustered genes of the well-characterized glucosinolate and flavonoid pathways in *A. thaliana*. Remarkably, we did not detect significant enrichment of H3K27me3 at these non-clustered natural product pathway genes compared to the genome-wide average of H3K27me3 markings (23.3 and 24.1% H3K27me3 enrichment at non-co-localized glucosinolate and flavonoid pathway genes c.f. 17% genome wide H3K27me3 enrichment; *P* = 0.24 and 0.21, respectively) (Supplementary Tables S2 and S3).

### Genome mining for H3K27me3-marked and co-expressed gene clusters

**Figure 4.**
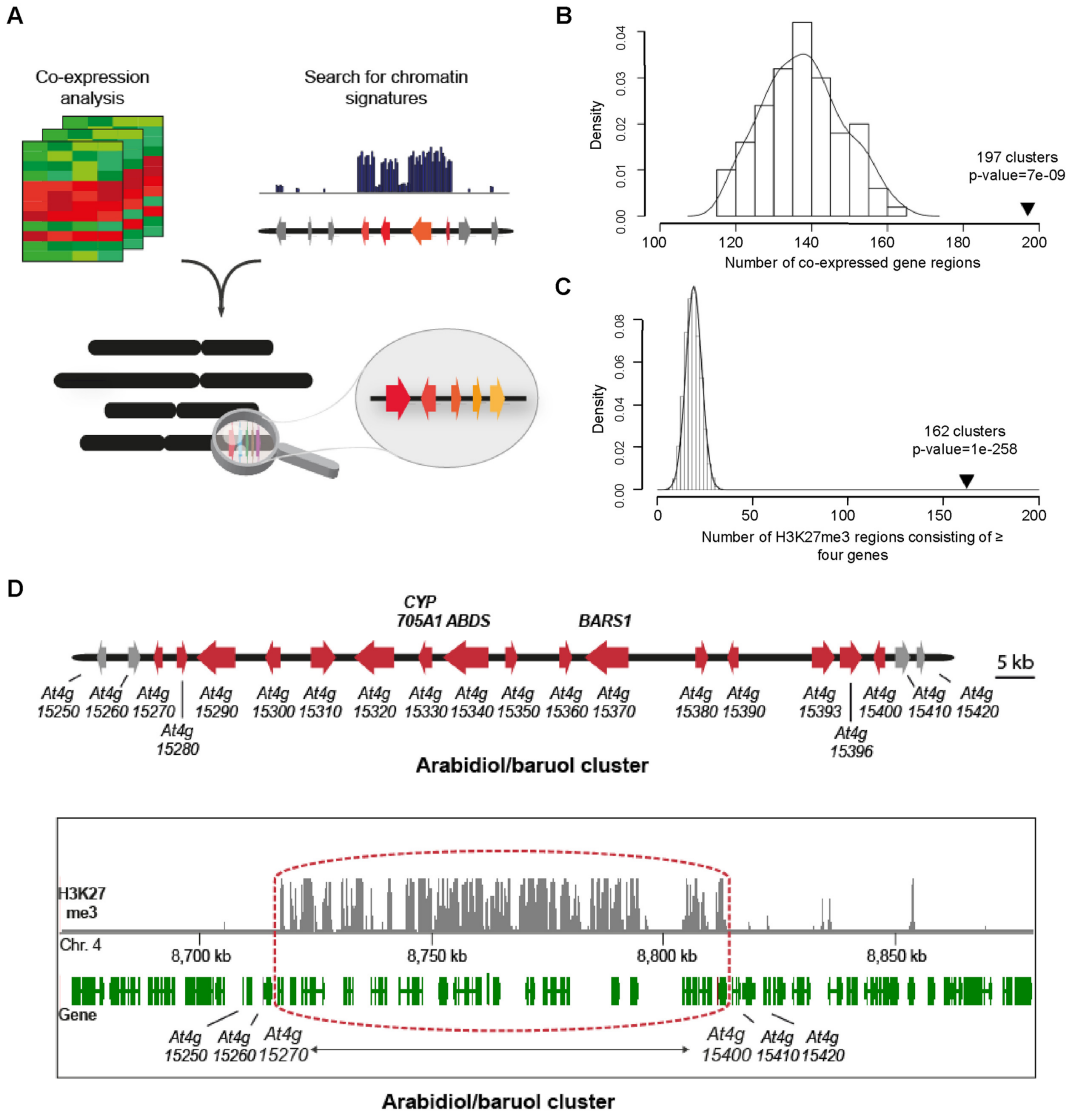
Genome mining for clusters of co-expressed and H3K27me3 marked genes. (**A**) Schematic showing the overall strategy used. Co-expressed genes were identified by analysis of genome-wide gene expression datasets and physically clustered gene sets within these identified. Groups of physically linked genes with contiguous H3K27me3 markings were identified by examination of genome-wide ChIP-on-chip datasets. Comparison of the outputs of these two analyses led to the identification of gene clusters that were both co-expressed and H3K27me3-marked. (**B**) Co-expressed gene regions in *Arabidopsis thaliana*. The high stringency maximal clique graph-based method for identification of co-localized and co-expressed gene clusters identified 197 regions (Supplementary Data Set 1; see ‘Materials and Methods’ section for details). Statistical significance was determined by randomly shuffling the gene order on each chromosome and reapplying the same methodological approach to find co-expressed clusters on the artificial chromosomes. After shuffling 100 times, the clique method gave a mean of 138 clusters with a standard deviation of 10. This resulted in a *P*-value of 7 × 10^−9^, assuming a normal distribution. (**C**) H3K27me3 marked regions of ≥ four genes in *A. thaliana*. 4629 of the 27 206 chromosomal protein coding genes in the *A. thaliana* genome were found to be H3K27me3 methylated (28). A search for four or more adjacent genes marked with H3K27me3 yielded 162 regions (Supplementary Data Set 2). To test for statistical significance of these regions, the methylation pattern was randomly shuffled using the Fisher-Yates shuffle algorithm. After 10 000 shuffles, the mean number of strings of four or more H3K27me3-marked genes obtained was 19.0 with a standard deviation of 4.2. This resulted in a *P*-value of 1 × 10^258^, assuming a normal distribution. (**D**) H3K27me3 markings at the arabidiol/baruol gene cluster. Top, the arabidiol/baruol cluster (red, cluster genes; grey, flanking genes). Below, ChIP-on-chip H3K27me3 marking at the *A. thaliana* arabidiol/baruol cluster [data extracted from Zhang *et al*. (28)]. The dataset (GSE7064) was uploaded with the University of California, Santa Cruz (UCSC) Genome Browser (University of California, Los Angeles installation). Genes are indicated in green; ChIP-on-chip marks are shown in grey. The cluster is framed in red.

Building on our finding that natural product biosynthetic gene clusters represent strings of contiguous genes that are strongly marked by H3K27me3, we carried out a genome-wide search in *A. thaliana* to search for co-ordinately expressed clusters of genes with H3K27me3 markings (see scheme in Figure 4A). To do this we analysed data from 1204 *A. thaliana* microarray experiments to identify groups of physically linked co-expressed genes, using a high-stringency maximal clique graph-based method (see ‘Materials and Methods’ section, Supplementary Data Set 1). In parallel we identified stretches of four or more contiguous H3K27me3-marked genes by mining the ChIP dataset from the Jacobsen lab (28) (Supplementary Data Set 2). Our analyses resulted in 197 clusters of co-localized and co-expressed genes and 162 clusters of adjacent H3K27me3 marked protein coding genes—significantly more clusters than expected in randomly shuffled artificial chromosomes (Figure 4B and C). We then compared the outputs of these two approaches to identify regions in common. We eliminated regions of tandem arrays that consisted of only one or two gene types in the process and set a minimum of at least three different gene family types required. This led us to identify seven H3K27me3-marked co-expressed regions, including the thalianol cluster, three other regions containing genes with known or predicted functions in natural product biosynthesis, and three regions harbouring genes with predicted functions in both metabolism and defence (Supplementary Table S4). One of the regions that we identified was a 16-gene cluster encoding enzymes for the synthesis and modification of arabidiol- and baruol-derived triterpene defence compounds (cluster #3; Supplementary Table S4), for which there is already some evidence of functional clustering (40,41). The striking localization of H3K27me3 markings to the genes in this cluster but not extending into the immediate flanking regions can be seen in Figure 4D. The functions of the other putative clusters are as yet unknown but, as for other plant specialized metabolic pathways, are likely to be associated with plant defence. The marneral cluster was not detected because part of our strategy involved looking for clusters of four or more H3K27me3-marked genes. However it was represented in a lower stringency co-expression dataset (Supplementary Figure S3; Supplementary Data Set 3).

By application of our co-expression protocol in maize we identified the DIMBOA cluster among 30 highly co-expressed regions (Supplementary Data Set 4). An overlay with the available genome-wide H3K27me3 map confirmed the overlap between the DIMBOA cluster co-expression region and H3K27me3 markings (Supplementary Table S5).

In further support of our cluster mining approach we analysed co-expression and H3K27me3 of the momilactone gene cluster in rice. Momilactones are diterpenes that have been shown to have functions in plant—plant allelopathy. The genes for their biosynthesis are clustered in the rice genome (9,42–43). We detected highly elevated co-expression values for all pairwise gene combinations in the cluster and all cluster genes show peaks of H3K27me3 in genome-wide H3K27me3 ChIPseq maps (Supplementary Table S6 and Supplementary Figure S5).

### The histone variant H2A.Z co-associates with metabolic gene clusters

**Figure 5.**
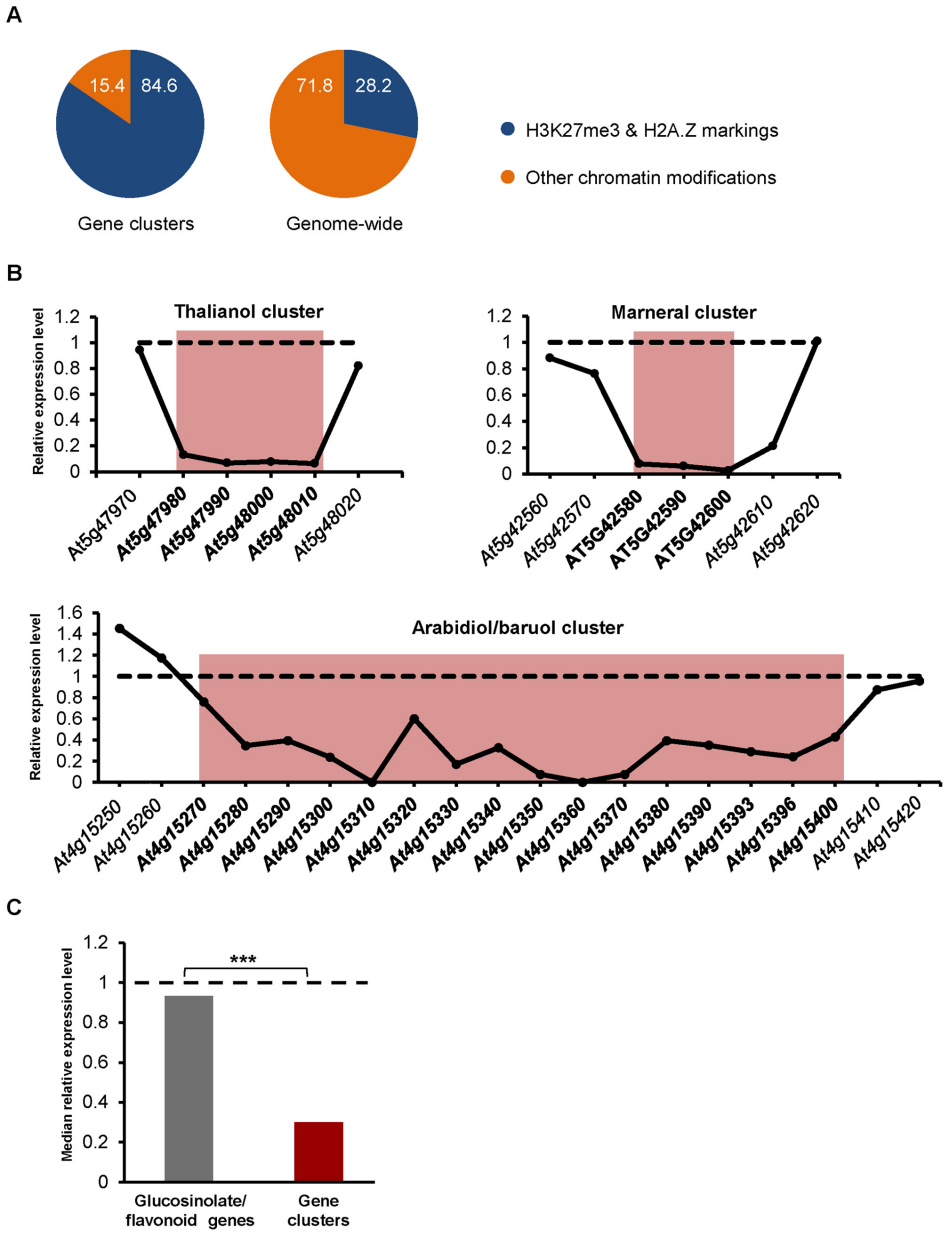
H3K27me3-marked co-expressed regions are marked by histone variant H2A.Z and show H2A.Z-dependent expression in *Arabidopsis thaliana*. (**A**) The seven *A. thaliana* clusters identified (Supplementary Table S4) are located in genomic regions that have just two predominant chromatin markings—H3K27me3 and H2A.Z. Data were extracted from a genome-wide chromatin map for *A. thaliana* that covers 16 different chromatin features (44) (Supplementary Figure S6; Supplementary Data Set 6). (**B**) Relative transcript levels of the thalianol, marneral and arabidiol/baruol cluster genes and flanking genes in the H2A.Z mutant line *hta9/11* (solid line) compared to the wild-type (dashed line) as assessed by RNAseq analysis. The cluster genes are indicated in bold and the cluster region highlighted in red. (**C**) Median relative expression values of genes for the seven *A. thaliana* clusters identified in Supplementary Table S4 and for the two well characterized non-clustered glucosinolate and flavonoid pathways in the H2A.Z mutant line *hta9/11*. The dashed line indicates wild-type expression. ***P < 0.001 (Mann–Whitney-U-Test).

To identify further chromatin markings that may also be involved in the delineation of metabolic gene clusters in plant genomes we analysed available data from a comprehensive genome-wide survey of *A. thaliana* chromatin states (44). The *A. thaliana* genome can be divided into regions typified by one of nine distinct chromatin states, where each state is characterized by a unique combination of chromatin modifications. Mapping of these chromatin states for the gene clusters identified above reveals prominent enrichment of only two chromatin states. Both states have just two characteristic chromatin features in common: H3K27me3 marking, which is in accordance with our cluster selection method, and increased H2A.Z deposition (Figure 5A; Supplementary Figure S6; Supplementary Data Set 6). We previously showed that the *A. thaliana* thalianol and marneral clusters have elevated H2A.Z levels in both genic and intergenic regions in tissues where the clusters are expressed, and that H2A.Z is required for cluster expression (Supplementary Figure S7) (45). We therefore carried out RNA-seq analysis of *A. thaliana* mutants with defective H2A.Z incorporation (*arp6, swc6* and *pie1*) or depleted H2A.Z levels (*hta9/11* double mutant) and searched the output for regions of three or more contiguous genes that showed coordinate effects on gene expression in at least three mutant backgrounds. Strikingly, all above identified gene clusters were strongly downregulated in the H2A.Z mutants, including the new arabidiol/baruol cluster (Figure 5B; Supplementary Table S7). This effect was cluster-specific and did not extend to include the immediate flanking genes (Figure 5B). In contrast, the expression levels of non-clustered metabolic pathway genes were not affected in H2A.Z mutants (Figure 5C; Supplementary Tables S8 and S9).

## DISCUSSION

The first plant metabolic gene cluster was reported in 1997 (7). However, it is only recently that an array of high impact publications has established that metabolic gene clusters are a widespread phenomenon in the plant kingdom (1,3). Genomic features that characterize such clusters of non-homologous pathway genes are scarce. Here, we show that two well-known chromatin marks, H2A.Z and H3K27me3, delineate metabolic gene clusters in *A. thaliana*. We further reveal that deposition of both marks is associated with changes in cluster expression. These hallmark chromatin marks can be used to mine plant genomes for cluster discovery, as we have demonstrated in *A. thaliana*.

In eukaryotes genomic co-localization of genes that belong to the same cellular process is best studied for clusters of tandemly duplicated genes. In animals it has been shown for the *Hox, β-globin* and *Irx* gene clusters that complex three-dimensional chromatin domains are formed at such clusters. It has been suggested that these domains separate gene clusters from the surrounding sequence space and enable efficient regulation (46–48). Histone modifications such as trimethylation of H3K27 have been linked to the formation of these domains (49,50). Similar chromatin domains have been reported at the human MHC locus which contains different types of genes co-localized at a single locus (51,52). We have previously shown by DNA fluorescence *in situ* hybridization (FISH) that the oat avenacin cluster undergoes chromatin rearrangements during the switch between transcribed and silenced state (53). Based on our data we speculate that H3K27me3 and H2A.Z are involved in a dynamic transition between different chromatin environments at highly localized and discrete genomic regions encompassing but not extending beyond the boundaries of plant metabolic gene clusters. Together these marks (along with other as yet unidentified factors) may facilitate highly restrictive regulation of cluster expression. Future experiments will investigate the interplay of H3K27me3 and H2A.Z marks at metabolic gene clusters to resolve the local and temporal order of these histone modifications. Chromosome conformation capture and super-resolution DNA-FISH experiments may also shed light on the changes in chromatin structure at clustered genes. Interestingly, genome binding studies of the PcG component LIKE HETEROCHROMATIN PROTEIN 1 (LHP1) showed an enrichment of LHP1 at tandemly duplicated genes in *A. thaliana* (54). This observation may indicate a wider role for a region-wide regulatory function of PcG in *A. thaliana*.

Our data presented here suggest that non-clustered metabolic pathway genes in *A. thaliana* are not significantly enriched for the predominant chromatin markings found at clustered pathway genes. Biosynthesis of glucosinolates and flavonoids are characterized by highly branched pathway networks with many enzymes required for the synthesis of more than one metabolite. In contrast, specialized metabolic pathways that are organized in clusters are mostly linear. It is conceivable that gene clustering and the type of higher level chromatin regulation reflect differences in the organizational form of metabolic pathways.

In contrast to the very recent discovery of plant metabolic gene clusters it has long been established that genomes of filamentous fungi harbour numerous clusters of biosynthetic pathway genes. Chromatin modifications have been identified that mark and regulate fungal clusters (55). The influence of these modifications on chromatin structure, however, remains largely unknown. Interestingly, manipulation of fungal chromatin regulatory processes has been shown to lead to the activation of clustered pathway genes, similarly to our observation of increased transcript levels for the thalianol and marneral clusters in *clf* mutant lines (56,57). Likewise, genome-wide histone modification analyses have indicated the precise delineation of fungal clusters by chromatin markings, as we have demonstrated here in plants (57). Medema *et al*. recently presented a genomic atlas of clustered metabolic pathways ranging from bacteria to fungi and for the first time also including plants (58). In the future epigenomic data could be incorporated into such maps to facilitate efficient delineation of eukaryotic clustered pathway genes.

## Supplementary Material

SUPPLEMENTARY DATA
