# Supplementary material for: Delineation of metabolic gene clusters in plant genomes by chromatin signatures
Source: Nucleic Acids Res. 2016 Feb 18;44(5):2255–65. doi: 10.1093/nar/gkw100 (PMC4797310; doi:10.1093/nar/gkw100)
Supplement: SUPPLEMENTARY DATA [file supp_44_5_2255__index.html]

Delineation of metabolic gene clusters in plant genomes by chromatin signatures — SUPPLEMENTARY DATA 

# Delineation of metabolic gene clusters in plant genomes by chromatin signatures

## SUPPLEMENTARY DATA

- SUPPLEMENTARY DATA
