# Supplementary material for: Delineation of metabolic gene clusters in plant genomes by chromatin signatures
Source: Nucleic Acids Res. 2016 Feb 18;44(5):2255–65. doi: 10.1093/nar/gkw100 (PMC4797310; doi:10.1093/nar/gkw100)
Supplement: SUPPLEMENTARY DATA [file supp_gkw100_nar-03298-h-2015-File.zip › Supplementary_Information_revision_170216.pdf]

Supplementary Information

Supplementary Figures 1-7, Supplementary Tables 1-10 and Supplementary script

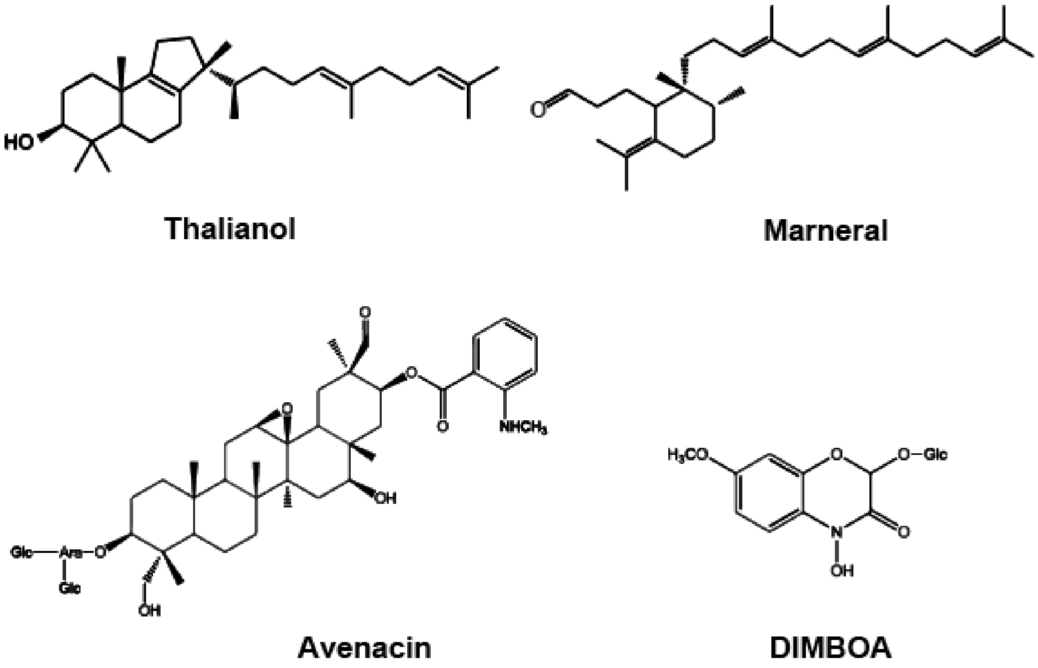

Supplementary Figure 1: Examples of metabolites produced by plant gene clusters.

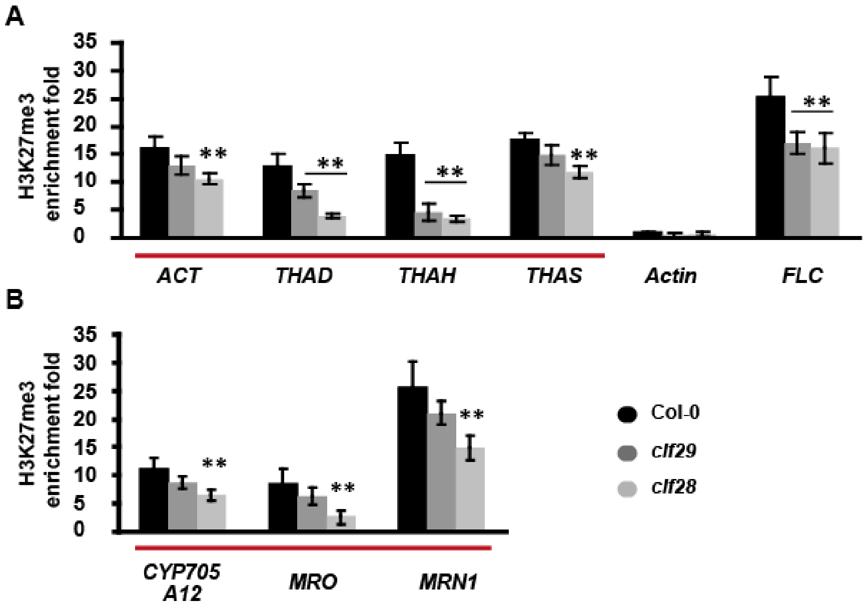

Supplementary Figure 2: H3K27me3 levels of the thalianol and marneral cluster genes in the wild type Col-0 line and in *clf29* and *clf28* mutants.

(**A**) H3K27me3 ChIP analysis of the thalianol cluster. Cluster genes are underlined in red. Controls included the *Actin* gene (*At3g18780*) (which has very low levels of H3K27me3) and *FLC* (a positive control).

(**B**) H3K27me3 ChIP analysis of the marneral cluster. Cluster genes are underlined in red. Experiments were carried out using six day-old seedlings.

Error bars indicate standard deviation of three biological replicates. **, P (*t*-test) < 0.01 mutant lines compared to wild type.

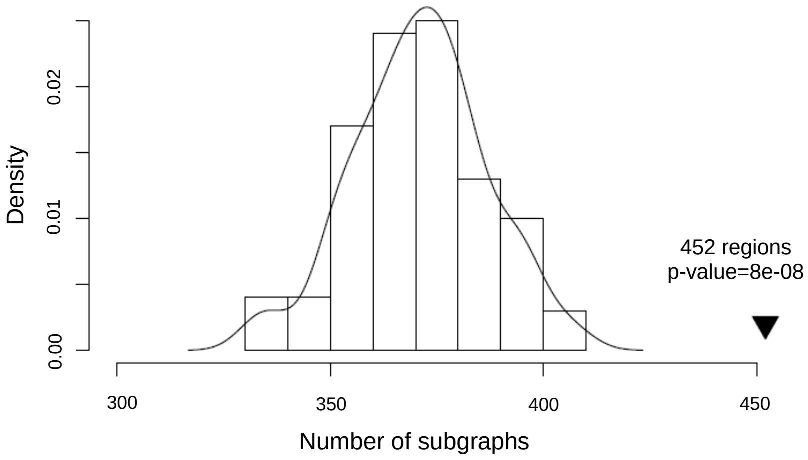

Supplementary Figure 3: Co-expressed gene regions in *A. thaliana* **–** subgraph method

The subgraph methods for finding co-localized co-expressed gene clusters resulted in 452 regions (see Methods for details). Statistical significance was determined by randomly shuffling the gene orders in each chromosome and reapplying the same methods to find co-expressed clusters in the new artificial chromosomes. After shuffling 100 times, the subgraph method resulted in a mean of 371 clusters (standard deviation 15, p-value of 8×10^{-8} assuming a normal distribution).

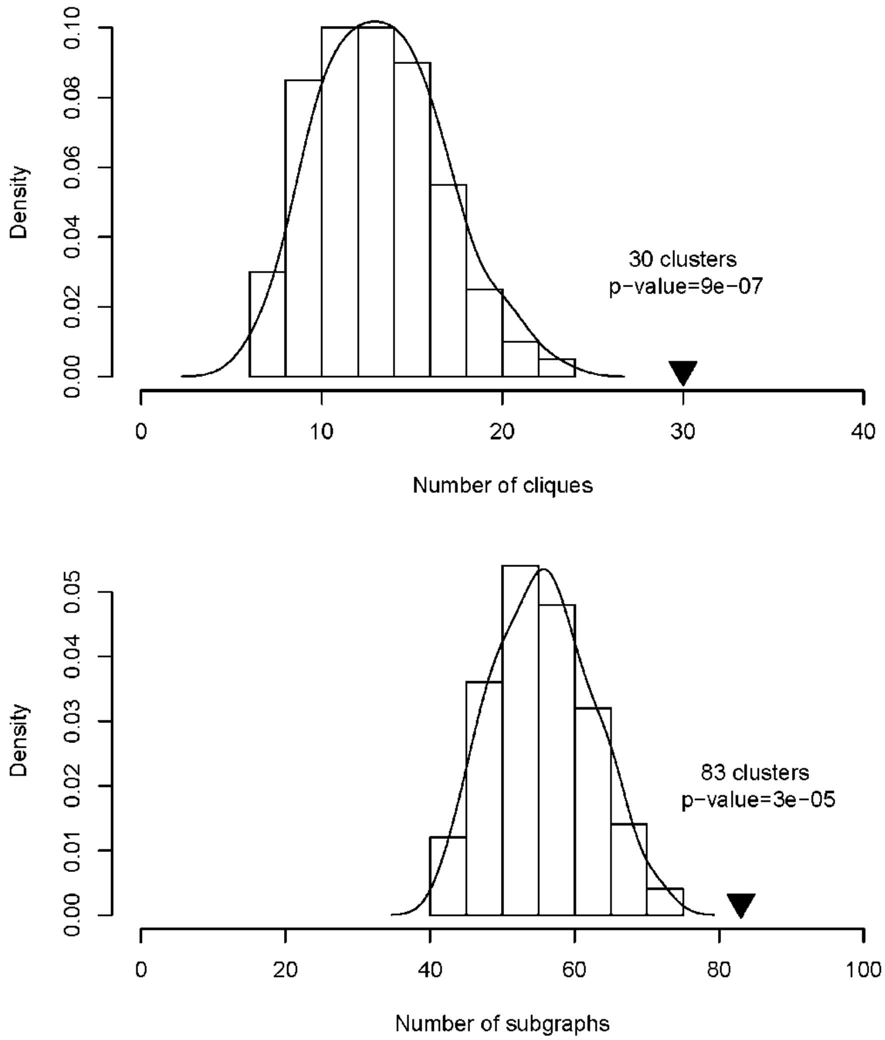

Supplementary Figure 4: Co-expressed gene regions in maize

The clique and subgraph methods for finding co-localized co-expressed gene clusters in maize resulted in 30 and 83 regions, respectively (see Methods for details). Statistical significance was determined by randomly shuffling the gene orders in each chromosome and reapplying the same methods to find co-expressed clusters in the new artificial chromosomes. After shuffling 100 times, both methods resulted in a mean of 13.38 (s.d. 3.48) and 55.65 (s.d. 6.78) clusters, respectively.

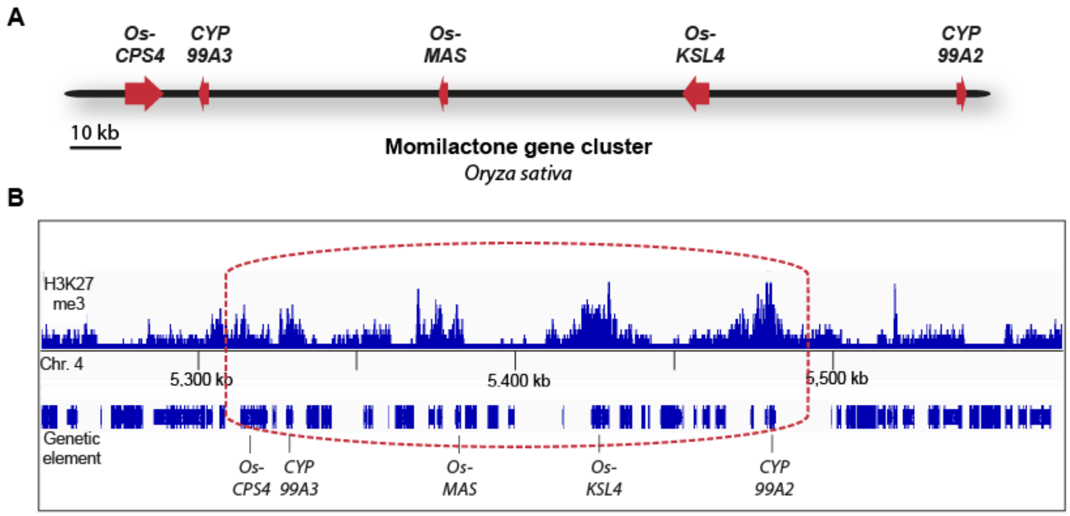

Supplementary Figure 5: Analysis of H3K27me3 markings at the rice momilactone cluster

(**A**) The *O. sativa* momilactone cluster.

(**B**) ChIP-seq data showing H3K27me3 marking at the momilactone cluster [data extracted from Hu et al. (59)]. The data set (GSM756376) was uploaded with the Integrative Genomics Viewer (Broad Institute). The momilactone gene cluster is framed in red.

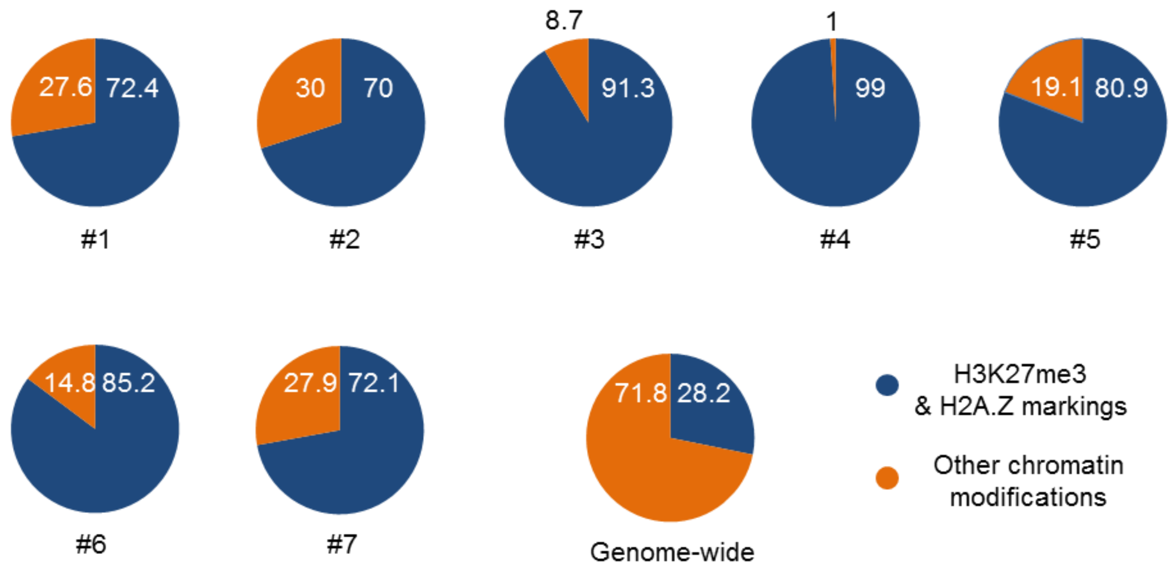

Supplementary Figure 6: Co-ordinate H3K27me3 and H2A.Z enrichment within gene clusters. The percentage of H3K27me3 and H2A.Z enriched regions within each of the seven clusters (as identified in Supplementary Table 6) compared to the whole genome are shown. The data is extracted from the *A. thaliana* genome wide chromatin maps generated by Sequeira-Mendes *et al.* (44), who mapped 16 different chromatin features (DNA methylation, histone modifications, histone variants) across the *A. thaliana* genome and reported nine different states that are characterized by distinct combinations of these chromatin features (Supplementary Data Set 6) (44). Two chromatin states are greatly enriched within the seven clusters identified above (Supplementary Data Set 6). Both chromatin states (states 4 and 5) have just two characteristic features in common: H3K27me3 marking, which is in accordance to our cluster selection method, and increased H2A.Z deposition (Supplementary Data Set 6).

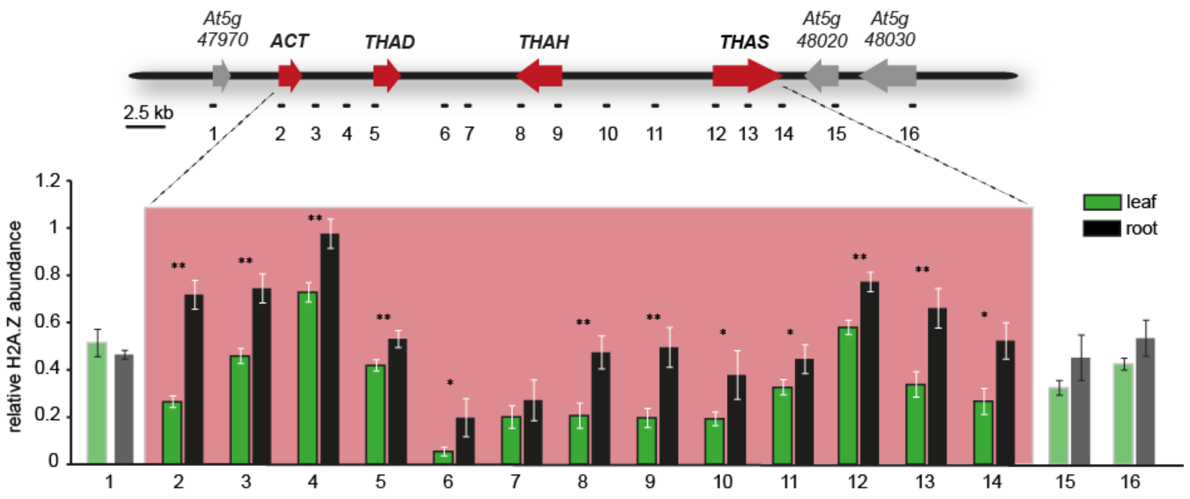

Supplementary Figure 7: Representation of H2A.Z deposition at thalianol cluster according to Nützmann and Osbourn (45).

H2A.Z ChIP analysis of the thalianol cluster is shown. Leaf (green bars) and root material (black bars) of six day-old seedlings of *A. thaliana* Col-0 were used. The cluster genes are indicated in bold and the cluster region highlighted in red. The analysed probes are indicated in the map of the thalianol cluster and below the histogram. Significantly increased H2A.Z levels were measured in roots compared to leaves inside the thalianol cluster. *, P (*t*-test) < 0.05, **, P (*t*-test) < 0.01.

Supplementary Table 1: Expression levels of the maize DIMBOA cluster genes in different tissues.

Data are extracted from Makarevitch et al. (39). Values indicate transcript values (reads per kilobase per million).

	**Tassel**	**Endosperm**	**Seedling**
Bx1	0.67	0.04	232.58
Bx2	15.36	0.02	88.80
Bx4	38.35	5.03	186.31
Bx5	72.73	17.01	272.56

Supplementary Table 2. H3K27me3 marking of glucosinolate pathway genes (60).

Gene	*Gene name*	*H3K27me3*	*Tandem duplicates*[‡]
At1g04580	AAO4		
At1g12140	FMO-GSOX5		
At1g16400	CYP79F2	+	*
At1g16410	CYP79F1	+	*
At1g18590	SOT17		
At1g24100	UGT74B1		
At1g31180	IPMDH3		
At1g62540	FMO-GSOX2	+	*
At1g62560	FMO-GSOX3	+	*
At1g62570	FMO-GSOX4	+	*
At1g65860	FMO-GSOX1	+	*
At1g65880	BZO1	+	*
At1g74090	SOT18		*
At1g74100	SOT16		*
At1g78370	GSTU20		
At2g14750	APK1		
At2g20610	SUR1		
At2g22330	CYP79B3	+	
At2g25450	GS-OH	+	
At2g30860	GSTF9		
At2g30870	GSTF10		
At2g31790	UGT74C1		
At2g43100	IPMI SSU2		
At3g03190	GSTF11		
At3g19710	BCAT4	+	
At3g39940	APK2		
At3g49680	BCAT3		
At3g58990	IPMI SSU3	+	
At4g03050	AOP3	+	*
At4g03060	AOP2		*
At4g12030	BAT5		
At4g13430	IPMI LSU1		
At4g13770	CYP83A1	+	
At4g23100	GSH1/PAD2		
At4g30530	GGP1		
At4g31500	CYP83B1		
At4g39950	CYP79B2	+	
At5g05260	CYP79A2	+	
At5g14200	IPMDH1		
At5g23010	MAM1	+	*

At5g23020	MAM3	+	*
At5g57220	CYP81F2		
At5g65940	CHY1		

[‡]Genes within a maximum distance of 10 gene regions and with BLASTn e-values of < 0.01 were designated tandem duplicates; +, H3K27me3 marking at respective genes according to Zhang et al. (2007) (28). 23.3 % of all non-clustered (excluding tandem duplicates) glucosinolate pathway genes are marked by H3K27me3 (cf 17 % H3K27me3 genome wide, $P = 0.24$, hypergeometric test). The entirety of glucosinolate pathway genes (including tandem duplicates) shows H3K27me3 markings at 39.5 % of genes ($P = 0.00038$, hypergeometric test, compared to genome wide H3K27me3)

Supplementary Table 3. H3K27me3 marking of flavonoid pathway genes (61).

Gene	*Gene name*	*H3K27me3*	*Tandem duplicates* [‡]
At1g03495	A3GlcCouT	+	
At1g03940			
At1g06000	F7RhaT (UGT89C1)		
At1g17260	AHA10		
At1g30530	F3RhaT (UGT78D1)		
At1g36160	ACC1		
At1g61720	ANR		
At1g65060	4CL3		
At2g23000	A3Glc2*XylSinT (SCPL10)		
At2g30490	C4H		
At2g36790	F7GlcT (UGT73C6)		
At2g37040	PAL1		
At3g29590	A5GlcMalT	+	
At3g51240	F3H		
At3g53260	PAL2		
At3g55120	CHI		
At3g59030	TT12		
At4g14090	A5GlcT (UGT75C1)		
At4g22880	LDOX/ANS		
At4g27830	BGLU10		
At5g05270			
At5g07990	F3'H (CYP75B1)	+	
At5g08640	FLS1	+	
At5g13930	CHS	+	
At5g17030	F3AraT (UGT78D3)	+	*
At5g17050	F3GlcT (UGT78D2)	+	*
At5g17220	GSTF12		
At5g42800	DFR	+	
At5g48100	LAC15	+	
At5g54060	A3G2*XylT (UGT79B1)		
At5g54160	OMT1		
At5g63590	FLS3	+	*

[‡]Tandem duplicates of genes as assessed by BLASTn e-values < 0.01 and maximum distance of 10 genes between each other; +, H3K27me3 marking at respective genes according to Zhang et al. (2007) (28). 24.1 % of all non-clustered (excluding tandem duplicates) flavonoid pathway genes are marked by H3K27me3 (cf 17 % H3K27me3 genome wide, P = 0.21, hypergeometric test). The entirety of flavonoid pathway genes (including tandem duplicates) shows H3K27me3 markings at 31.25 % of genes (P = 0.035, hypergeometric test, compared to genome wide H3K27me3).

Supplementary Table 4. Clusters of H3K27me3-marked genes from Supplementary Data Set 2 that were also identified in the high stringency co-expression analysis (Supplementary Data Set 1).

Cluster	*Gene*	*Predicted function*	*H3K27 me3*	*Clique no*
**#1**	*At2g17050*	TIR-NBS-LRR class disease resistance protein [Source:EMBL;Acc:AEC06580.1]	+	27
	At2g17055	Toll-Interleukin-Resistance (TIR) domain family protein [Source:EMBL;Acc:AEC06581.1]	+	
	At2g17060	TIR-NBS-LRR class disease resistance protein [Source:EMBL;Acc:AEC06582.1]	+	27
	At2g17070	uncharacterized protein [Source:EMBL;Acc:AEC06583.1]	+	27
	At2g17080	uncharacterized protein [Source:EMBL;Acc:AEC06584.1]		27
	At2g17150	RWP-RK domain-containing protein [Source:EMBL;Acc:AEC06592.1]		27
**#2**	*At3g01190*	peroxidase 27 [Source:EMBL;Acc:AEE73623.1]	+	31
	At3g01220	homeobox-leucine zipper protein ATHB-20 [Source:EMBL;Acc:AEE73626.1]	+	31
	At3g01230	uncharacterized protein [Source:EMBL;Acc:AEE73627.1]	+	
	At3g01240	uncharacterized protein [Source:EMBL;Acc:AEE73628.1]	+	
	At3g01250	uncharacterized protein [Source:EMBL;Acc:AEE73629.1]	+	
	At3g01260	aldose 1-epimerase domain-containing protein [Source:EMBL;Acc:AEE73630.1]	+	31
	At3g01270	putative pectate lyase 7 [Source:EMBL;Acc:AEE73631.1]	+	
	At3g01280	mitochondrial outer membrane protein porin 1 [Source:EMBL;Acc:AEE73632.1]		31
**#3**	*At4g15270*	glucosyltransferase-related protein [Source:EMBL;Acc:AEE83579.1]	+	1
	At4g15280	UDP-glucosyl transferase 71B5 [Source:EMBL;Acc:AEE83580.1]	+	
	At4g15290	cellulose synthase-like protein B5 [Source:EMBL;Acc:AEE83581.1]	+	
**Arabidiol/**	*At4g15300*	cytochrome P450, family 702, subfamily	+	1

**baruol cluster**		A, polypeptide 2 [Source:EMBL;Acc:AEE83582.1]		
	At4g15310	cytochrome P450, family 702, subfamily A, polypeptide 3 [Source:EMBL;Acc:AEE83583.1]	+	
	At4g15320	cellulose synthase-like protein B6 [Source:EMBL;Acc:AEE83584.1]	+	
	At4g15330	cytochrome P450, family 705, subfamily A, polypeptide 1 [Source:EMBL;Acc:AEE83585.1]	+	1
	At4g15340	arabidiol synthase [Source:EMBL;Acc:AEE83586.1]	+	1
	At4g15350	cytochrome P450, family 705, subfamily A, polypeptide 2 [Source:EMBL;Acc:AEE83587.1]	+	1
	At4g15360	cytochrome P450, family 705, subfamily A, polypeptide 3 [Source:EMBL;Acc:AEE83588.1]	+	1
	At4g15370	baruol synthase [Source:EMBL;Acc:AEE83589.1]	+	1
	At4g15380	cytochrome P450, family 705, subfamily A, polypeptide 4 [Source:EMBL;Acc:AEE83590.1]	+	1
	At4g15390	HXXXD-type acyl-transferase family protein [Source:EMBL;Acc:AEE83591.1]	+	1
	At4g15393	cytochrome P450, family 702, subfamily A, polypeptide 5 [Source:EMBL;Acc:AEE83594.1]	+	
	At4g15396	cytochrome P450, family 702, subfamily A, polypeptide 6 [Source:EMBL;Acc:AEE83595.1]	+	
	At4g15400	HXXXD-type acyl-transferase-like protein [Source:EMBL;Acc:AEE83596.1]	+	1
**#4**	*At5g15100*	putative auxin efflux carrier component 5 [Source:EMBL;Acc:AED92117.1]	+	79
	At5g15110	putative pectate lyase 19 [Source:EMBL;Acc:AED92118.1]	+	79
	At5g15130	putative WRKY transcription factor 72 [Source:EMBL;Acc:AED92120.1]	+	
	At5g15140	aldose 1-epimerase [Source:EMBL;Acc:AED92121.1]	+	79
	At5g15150	homeobox-leucine zipper protein HAT7 [Source:EMBL;Acc:AED92122.1]	+	
	At5g15160	protein banquo 2 [Source:EMBL;Acc:AED92123.1]	+	
**#5**	*At5g37940*	2-alkenal reductase [Source:EMBL;Acc:AED94249.1]	+	
	At5g37950	UDP-glycosyltransferase-like protein [Source:EMBL;Acc:AED94251.1]	+	

	At5g37960	GroES-like family protein [Source:EMBL;Acc:AED94252.1]	+	
	At5g37970	S-adenosyl-L-methionine-dependent methyltransferase-like protein [Source:EMBL;Acc:AED94253.1]	+	
	At5g37980	2-alkenal reductase [Source:EMBL;Acc:AED94254.1]	+	
	At5g37990	S-adenosyl-L-methionine-dependent methyltransferase-like protein [Source:EMBL;Acc:AED94255.1]	+	190
	At5g38000	2-alkenal reductase [Source:EMBL;Acc:AED94256.1]	+	
	At5g38010	UDP-glycosyltransferase-like protein [Source:EMBL;Acc:AED94257.1]	+	
	At5g38020	S-adenosyl-L-methionine-dependent methyltransferase-like protein [Source:EMBL;Acc:AED94258.1]	+	190
	At5g38030	mate efflux domain-containing protein [Source:EMBL;Acc:AED94259.1]	+	190
**#6**	*At5g47950*	HXXXD-type acyl-transferase-like protein [Source:EMBL;Acc:AED95598.1]		10
	At5g47980	BAHD acyltransferase [Source:EMBL;Acc:AED95602.1]	+	10
**Thalianol cluster**	***At5g47990***	cytochrome P450 705A5 [Source:EMBL;Acc:AED95603.1]	+	10
	At5g48000	cytochrome P450 708A2 [Source:EMBL;Acc:AED95608.1]	+	10
	At5g48010	thalianol synthase 1 [Source:EMBL;Acc:AED95609.1]	+	10
	At5g48070	xyloglucanendotransglucosylase/ hydrolase protein 20 [Source:EMBL;Acc:AED95616.1]		10
**#7**	*At5g63560*	HXXXD-type acyl-transferase-like protein [Source:EMBL;Acc:AED97769.1]	+	19
	At5g63580	flavonol synthase 2 [Source:EMBL;Acc:AED97771.1]	+	
	At5g63590	flavonol synthase 3 [Source:EMBL;Acc:AED97772.1]	+	19
	At5g63595	flavonol synthase 4 [Source:EMBL;Acc:AED97773.1]	+	
	At5g63600	flavonol synthase 5 [Source:EMBL;Acc:AED97775.1]	+	19
	At5g63650	serine/threonine-protein kinase SRK2H [Source:EMBL;Acc:AED97781.1]		19
	At5g63660	defensin-like protein 6 [Source:EMBL;Acc:AED97782.1]		19

+, H3K27me3 marking (Supplementary Data Set 2); Clique numbers indicate genes identified as being co-expressed in the maximal clique co-expression data set (Supplementary Data Set 1). The

thalianol cluster genes are highlighted in bold. Two genes that do not immediately flank this cluster but are closely linked to it were also identified as being co-expressed (*At5g47950* and *At5g48070*).

Supplementary Table 5. The maize DIMBOA cluster identified by cluster mining.

Cluster	***Gene***	***Predicted function***	***H3K27 me3***	***Clique no***
**DIMBOA**	*GRMZM2G172491*	3-hydroxyindolin-2-one monooxygenase [Source:UniProtKB/Swiss-Prot;Acc:Q43250]	+	5
	GRMZM2G063756	Cytochrome P450 71C3 [Source:UniProtKB/Swiss-Prot;Acc:P93703]	+	5
	GRMZM2G085054	DIMBOA UDP-glucosyltransferase BX8 [Source:UniProtKB/Swiss-Prot;Acc:Q8W2B7]		
	GRMZM2G085303	Uncharacterized protein [Source:UniProtKB/TrEMBL;Acc:K7TQR3]		
	GRMZM5G856095	Uncharacterized protein [Source:UniProtKB/TrEMBL;Acc:K7TUD8]		
	GRMZM2G085381	Indole-3-glycerol phosphate lyase, chloroplastic [Source:UniProtKB/Swiss-Prot;Acc:P42390]	+	5
	GRMZM2G085661	Indole-2-monooxygenase [Source:UniProtKB/Swiss-Prot;Acc:Q43257]	+	5

Supplementary Table 6: Co-expression values of momilactone cluster genes.

Pearson correlation coefficients were calculated for each gene with GENEVESTIGATOR based on set of 2532 samples (62).

	OsCPS4	***CYP99A3***	***OsMas***	***OsKSL4***	***CYP99A2***
OsCPS4		0.69*	0.74*	0.78*	0.82*
CYP99A3	0.69*		0.53	0.54	0.66*
OsMAS	0.74*	0.53*		0.74*	0.8*
OsKSL4	0.78*	0.54*	0.74*		0.85*
CYP99A2	0.82*	0.66*	0.8*	0.85*	

*Co-expression values within the top 15 co-expressed genes.

Supplementary Table 7. Relative expression of the seven gene clusters in the Col-0 wild type and H2A.Z (*hta9/11*) and SWR1 mutants (*arp6*, *swc6*, *pie1*).

Cluster	*Gene*	Col-0	*hta9/11*	*arp6*	*swc6*	*pie1*
	At2g17040	*1*	*1.834*	*1.9062*	*3.1388*	*1.4244*
**#1**	**At2g17050**	1	0.4387	0.1792	0.6468	1.3131
	**At2g17055**	1	0.0906	0.1803	0.1587	0.5997
	**At2g17060**	1	0.3344	0	0.429	1.2074
	**At2g17070**	1	0.1385	0.6471	0	1.1174
	**At2g17080**	1	0.4298	0.6095	0.6663	1.1017
	**At2g17150**	1	0.9091	0.7285	0.7978	0.7521
	At2g17180	*1*	*0.7636*	*0.8465*	*0.9866*	*0.8574*
	At3g01180	*1*	*1.08*	*1.0644*	*1.1829*	*1.1114*
**#2**	**At3g01190**	1	0.311	0.3885	0.6142	1.2614
	**At3g01220**	1	0.4612	0.415	0.5901	0.9279
	**At3g01230**	n/a	n/a	n/a	n/a	n/a
	**At3g01240**	n/a	n/a	n/a	n/a	n/a
	**At3g01250**	n/a	n/a	n/a	n/a	n/a
	**At3g01260**	1	0.5141	1.2081	0.6265	2.2032
	**At3g01270**	n/a	n/a	n/a	n/a	n/a
	**At3g01280**	1	0.9932	1.1906	1.1032	1.3776
	At3g01290	*1*	*1.9046*	*0.863*	*1.2911*	*0.9423*
	At4g15250	*1*	*1.2079*	*1.4535*	*0*	*1.2727*
	At4g15260	*1*	*1.278*	*1.1748*	*1.2106*	*1.1082*
**#3**	**At4g15270**	1	0.761	0.7094	0.6250	1.8604
	**At4g15280**	1	0.3467	0.7362	1.0013	8.597
	**At4g15290**	1	0.3937	0.4516	0.5960	0.6016
**Arabidiol/**	**At4g15300**	1	0.2373	0.2027	0.6537	0.9387
**baruol**	**At4g15310**	1	0	1.1257	0	5.6511
**cluster**	**At4g15320**	1	0.6013	0.9596	0.7146	0.7419
	**At4g15330**	1	0.1712	0.221	0.364	1.2571
	**At4g15340**	1	0.3269	0.302	0.3263	1.7151
	**At4g15350**	1	0.0755	0.219	0.3591	1.5801
	**At4g15360**	1	0	0.2515	0.8092	3.538
	**At4g15370**	1	0.0754	0.065	0.1456	1.0428
	**At4g15380**	1	0.3945	0.1693	0.2974	0.747
	**At4g15390**	1	0.3497	0.3371	0.4132	1.5985
	**At4g15393**	1	0.2896	0.237	0.2846	1.2218
	**At4g15396**	1	0.2407	0.0688	0.3821	1.1739
	**At4g15400**	1	0.429	0.4997	0.6304	0.8941
	At4g15410	*1*	*0.858*	*0.8742*	*1.0622*	*0.9539*
	At4g15415	*1*	*1.0203*	*0.9563*	*1.0017*	*0.9829*

	At5g15090	*1*	*1.1783*	*1.1125*	*1.0876*	*1.1635*
#4	**At5g15100**	1	0.4795	0.4935	0.9954	0.911
	**At5g15110**	1	0	0	0	0
	**At5g15130**	1	0.3008	0.268	0.5080	1.211
	**At5g15140**	n/a	n/a	n/a	n/a	n/a
	**At5g15150**	1	0.4736	0.7961	0.5819	1.0121
	**At5g15160**	1	0.7492	0.7177	0.9698	0.6272
	At5g15190	*1*	*1.6753*	*2.4359*	*1.607*	*1.8893*
	At5g37930	1	1.1376	1.0838	0.9346	1.0178
#5	**At5g37940**	1	0.5392	0.6354	0.5395	0.9276
	**At5g37950**	1	0.2097	1.0334	0.4392	0.983
	**At5g37960**	1	0.29	0.3997	0	0.3935
	**At5g37970**	1	0	0.1737	0.1243	0.3988
	**At5g37980**	1	0.6826	0.7168	0.9454	1.6469
	**At5g37990**	1	0.1848	0.1376	0.2835	0.3716
	**At5g38000**	1	0.4257	0.3153	0.5396	0.6803
	**At5g38010**	1	0.2675	0.349	0.3790	0.7519
	**At5g38020**	1	0.1853	0.0912	0.2397	0.7129
	**At5g38030**	1	0.2262	0.2141	0.3245	0.7067
	At5g38040	*1*	*0.3417*	*0.5227*	*0.1763*	*0.3202*
	At5g38050	*1*	*0.9128*	*0.819*	*0.3376*	*0.7723*
	At5g47930	*1*	*1.0541*	*1.2079*	*1.1791*	*1.2532*
#6	*At5g47940*	*1*	*0.4879*	*0.7529*	*1.1005*	*0.7802*
	**At5g47950**	1	0.2566	0.2107	0.3341	0.9474
**Thalianol**	**At5g47980**	1	0.132	0.689	0.2495	1.0489
**cluster**	**At5g47990**	1	0.0672	0.0678	0.1189	0.4186
	**At5g48000**	1	0.0779	0.0766	0.1089	0.2819
	**At5g48010**	1	0.063	0.036	0.0778	0.2793
	**At5g48070**	1	0.2761	0.3454	0.5478	2.8404
	At5g48090	*1*	*6.1724*	*1.3011*	*0.8331*	*1.1423*
	At5g63550	*1*	*0.9445*	*0.835*	*0.7435*	*0.7958*
#7	**At5g63560**	1	0.2945	0.3373	0.5213	1.0217
	**At5g63580**	1	2.1384	2.0165	3.6929	6.4761
	**At5g63590**	1	0.3761	0.4178	0.5733	1.0644
	**At5g63595**	1	0.3582	0.374	0.5010	0.6364
	**At5g63600**	1	0.3821	0.3472	0.4738	1.3192
	**At5g63650**	1	0.6913	1.0582	0.9798	1.3886
	**At5g63660**	1	0.1784	0.5402	0.5242	0.9016
	At5g63670	*1*	*1.1787*	*0.9984*	*0.905*	*1.0171*

Relative expression value

0.15 1 6

Changes in gene expression relative to wild type Col-0 were calculated using the RPKM values of the respective mutants. Relative expression values are colour coded to visualize the direction and magnitude of misregulation: yellow, no change; green, up-regulated; red, down-regulated. Cluster genes are shown in bold and flanking genes in italics. Comparison of genome-wide and cluster-wide down-regulation of gene transcript levels (≥ 2-fold down-regulation) in *hta9/hta11, arp6* and *swc6* showed highly significant enrichment within the gene clusters (5.47 % genome-wide cf 81.48 % cluster-wide downregulation in *hta9/hta11*, $P(X \geq 44) = 2.49 \times 10^{-46}$; 6.41 % cf 66.6 % in *arp6*, $P(X \geq 36) = 2.66 \times 10^{-30}$; 3.92% cf 48.14 % in *swc6*, $P(X \geq 26) = 1.4 \times 10^{-22}$, hypergeometric test).

Supplementary Table 8. Relative expression of glucosinolate biosynthesis genes (60) in the Col-0 wild type and H2A.Z (*hta9/11*) and SWR1 (*arp6*, *swc6*, *pie1*) mutants.

Gene	*Gene name*	Col-0	*hta9/11*	*arp6*	*swc6*	*pie1*
At1g04580	AAO4	1	1.5883	1.5649	2.9153	12.1576
At1g12140	FMO-GSOX5	1	1.0611	1.164	1.2136	1.62899
At1g16400	CYP79F2	1	1.0692	0.8944	1.0113	1.37774
At1g16410	CYP79F1	1	0.7992	0.764	0.8758	1.28987
At1g18590	SOT17	1	0.9537	1.0274	1.106	2.21639
At1g24100	UGT74B1	1	0.7736	0.9623	0.852	1.53252
At1g31180	IPMDH3	1	1.2179	1.2882	1.3084	1.04202
At1g62540	FMO-GSOX2	1	0.8994	1.0014	1.0807	3.92829
At1g62560	FMO-GSOX3	1	0.9445	1.1596	0.9937	1.36056
At1g62570	FMO-GSOX4	1	0.7636	1.0056	0.9682	1.69593
At1g65860	FMO-GSOX1	1	0.7206	0.8154	0.7899	1.58018
At1g65880	BZO1	1	2.5783	0.7986	1.8789	21.0713
At1g74090	SOT18	1	0.854	0.9323	0.927	1.32511
At1g74100	SOT16	1	0.752	1.0685	0.9209	2.46163
At1g78370	GSTU20	1	0.9374	1.0473	1.0243	1.37936
At2g14750	APK1	1	0.8749	1.1873	0.9054	2.09461
At2g20610	SUR1	1	0.8395	0.9782	0.9266	1.51633
At2g22330	CYP79B3	1	0.4204	0.4127	0.4557	3.04772
At2g25450	GS-OH	1	1.1303	0.3086	1.1514	1.47812
At2g30860	GSTF9	1	0.6866	0.9719	0.823	1.68108
At2g30870	GSTF10	1	0.825	1.2136	0.9416	2.2042
At2g31790	UGT74C1	1	1.1348	1.2618	1.1643	1.29811
At2g43100	IPMI SSU2	1	0.9447	1.1714	1.0555	1.16619
At3g03190	GSTF11	1	0.882	0.8928	1.0205	1.50757
At3g19710	BCAT4	1	0.9006	0.9066	1.0001	1.85176
At3g39940	APK2	n/a	n/a	n/a	n/a	n/a
At3g49680	BCAT3	1	0.9054	1.0488	0.9318	0.83091
At3g58990	IPMI SSU3	1	0.8973	1.146	0.9786	1.33994
At4g03050	AOP3	1	3.9135	8.9806	2.1562	0.8552
At4g03060	AOP2	1	0.7748	1.1248	0.847	0.78576
At4g12030	BAT5	1	0.8181	0.8035	0.8222	1.06046
At4g13430	IPMI LSU1	1	0.7916	0.8378	0.8136	1.08113
At4g13770	CYP83A1	1	0.9311	1.0023	1.0098	1.45014
At4g23100	GSH1/PAD2	1	0.9122	0.9541	0.9476	1.44148
At4g30530	GGP1	1	1.0337	1.2608	1.0662	2.8662
At4g31500	CYP83B1	1	0.5748	0.8127	0.6745	2.52062

At4g39950	CYP79B2	1	0.389	0.4925	0.4782	2.55817
At5g05260	CYP79A2	1	0	1.5498	0	1.09445
At5g14200	IPMDH1	1	0.909	0.9633	0.9912	1.50161
At5g23010	MAM1	1	0.84	0.8313	0.8005	1.17921
At5g23020	MAM3	1	0.7173	0.8335	1.0412	2.66155
At5g57220	CYP81F2	1	0.9452	1.1689	1.1924	2.09878
At5g65940	CHY1	1	1.1414	1.1766	1.2512	1.18726

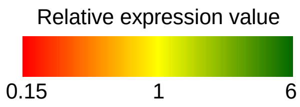

Changes in gene expression relative to Col-0 wild type are calculated using the RPKM values of the respective mutants. Relative expression values are colour-coded to visualize the direction and magnitude of misregulation: yellow, no change; green, up-regulated; red, down-regulated.

Supplementary Table 9. Relative expression of flavonoid biosynthesis genes (61) in Col-0 wild type and H2A.Z (*hta9/11*) and SWR1 (*arp6*, *swc6*, *pie1*) complex mutants.

Gene	*Gene name*	Col-0	*hta9/11*	*arp6*	*swc6*	*pie1*
At1g03495	A3GlcCouT	1	1.4337	1.2401	1.2865	1.2309
At1g03940	AT1G03940	1	1.2897	0.5543	1.229	1.4501
At1g06000	F7RhaT (UGT89C1)	1	0.8524	1.2168	0.8763	2.485
At1g17260	AHA10	1	0.4067	0.4559	0.4932	0.3869
At1g30530	F3RhaT (UGT78D1)	1	1.4123	1.5123	1.2916	3.0133
At1g36160	ACC1	1	1.0283	0.9378	1.0634	1.0475
At1g61720	ANR	1	1.1897	0.4718	0	1.4419
At1g65060	4CL3	1	1.2225	0.9518	1.0498	1.5345
At2g23000	A3Glc2*XylSinT (SCPL10)	1	1.2305	1.406	1.0195	1.1234
At2g30490	C4H	1	1.0841	0.7913	1.1137	1.7132
At2g36790	F7GlcT (UGT73C6)	1	0.9855	0.8014	1.0561	1.4539
At2g37040	PAL1	1	0.9906	0.8999	1.1143	2.3448
At3g29590	A5GlcMalT	1	0.5289	0.6459	0.5511	3.2707
At3g51240	F3H	1	1.7756	1.3408	1.4918	1.7546
At3g53260	PAL2	1	1.0498	0.9142	1.0466	1.4249
At3g55120	CHI	1	1.7365	1.6445	1.6898	1.8904
At3g59030	TT12	1	0.5767	0.3318	0.8272	0.1935
At4g14090	A5GlcT (UGT75C1)	1	0.8973	0.9528	0.9149	4.1356
At4g22880	LDOX/ANS	1	0.6006	0.6764	0.4568	4.6771
At4g27830	BGLU10	1	1.0675	0.9838	1.0259	0.9048
At5g05270		1	1.2766	1.5874	1.1674	1.704
At5g07990	F3'H (CYP75B1)	1	1.5154	0.97	1.0303	1.9087
At5g08640	FLS1	1	1.0643	1.3326	1.208	1.8246
At5g13930	CHS	1	1.298	1.0747	1.0133	1.6719
At5g17030	F3AraT (UGT78D3)	1	12.406	19.89	10.295	17.226
At5g17050	F3GlcT (UGT78D2)	1	1.0594	1.0766	1.1704	1.6334
At5g17220	GSTF12	1	0.9643	0.7942	0.8039	4.6575
At5g42800	DFR	1	0.4871	0.5171	0.4225	5.1493
At5g48100	LAC15	1	0.1906	0.314	0.3472	0.5956
At5g54060	A3G2*XylT (UGT79B1)	1	0.6002	0.6012	0.4652	6.2913
At5g54160	OMT1	1	1.2403	1.0323	1.1055	1.5535
At5g63590	FLS3	1	0.3761	0.4178	0.5733	1.0644

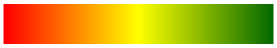

Changes in gene expression relative to Col-0 wild type are calculated using the RPKM values of the respective mutants. Relative expression values are colour-coded to visualize the direction and magnitude of misregulation: yellow, no change; green, up-regulated; red, down-regulated.

Supplementary Table 10: Oligonucleotide sequences used in this work.

**Oligos**	**Sequence 5'-3'**
qRT	
47980qRT-F	CGGCCATACTAACACTACACTT
47980qRT-R	TGCCTACCCATACTTCAACAC
47990qRT-F	ATCCTGATTTCTGGGAAGACC
47990qRT-R	TGTTGCACCATCATTCCAATT
48000qRT-F	TGGTGTTTGGAGGTGGAGTGA
48000qRT-R	GGGAAATCTTGATAGGCAGTC
48010qRT-F	CTTCAATCCACTATGGCACTC
48010qRT-R	TAAAATAATCACTCTTAGGGTCTTC
atCAS-qRT-F	GAAAGGACGAGACTGGATACTAAA
atCAS-qRT-R	TACAAATACGACATCGGCAAG
qat5g48020-F	TTACAACGAGGAAGGGACGAT
qat5g48020-R	ACCGGAACTTTAACCGCAACC
qRT-42580-F	GTCTCGTATCGCTTTGTCCCA
qRT-42580-R	CTTCTTCCTCGCCTTACCTTG
qRT-42590-F	ATCACGAAATCTGCCCATAAA
qRT-42590-R	CTTCCAGCGTCTCCATCAATA
qRT-42600-F	ATGCTTGCTTGTTGGGTAGAA
qRT-42600-R	TTTGCGGCTAACATAACTTGG
qat5g42570-F	GGTTTCTTATGGAGTATGATAGGTT
qat5g42570-R	TTCTTTCCCTCAGGACTATGC
GAPDHq-5'	TGTTGAGGGTCTGATGACCA
GAPDHq-3'	TGCTTGGGAATGATGTTGAA
ChIP	
FLC-F	CGGTCTCATCGAGAAAGCTC
FLC-R	CCACAAGCTTGCTATCCACA
H3K27-48020-F1	ACAATGCCTAATGAATACAAACCAC
H3K27-48020-R1	ATCCGAAGATCAAACCTTAAACC
H3K27-42570-F1	TGATCCTTCTCCTCCTCTTCA
H3K27-42570-R1	ATACGGTGGTTCCTATGGTTT
K27-47980-N3-F	CGGCCATACTAACACTACACTTG
K27-47980-N3-R	CCATCCGAAATCGACCTCATA
K27-47990-N3-F	GAGGAGAACGGTGAGGCTGAG

K27-47990-N3-R	TGAAACATGGCTCCCAAGAAA
k27-48000-gene-F	TTATTCAAACTTCAAAGGCAAAACC
k27-48000-gene-R	TAATCACAACAGCGGCTACAGCTAT
k27-48010-gene-F	AACAGAAGATTCCACGAGTGATAA
k27-48010-gene-R	GCAAGGCTGTGAAATAGAGTAAC
h3k27-42580new2-F	CCACGAAACTACTCCGACCAC
h3k27-42580new2-R	CTTCTTCCTCGCCTTACCTTG
h3k27-42590new1-F	TCATTGGAAACCTCCATCAGC
h3k27-42590new1-R	AAACGAAGGAGCATGAGTGGC
h3k27-42600new4-F	ACGGTCCTCAACTACATCTGC
h3k27-42600new4-R	AAGCTTTAGCCACCATAGGCG
H3K27-SAD1g-F	ATAGATAATTGGCGTTTACGAATGG
H3K27-SAD1g-R	TACCTGGGTGAATCGGAAGAA
H3K27-SAD2g-F	ATTGCTGGTCGCTGCCTACTT
H3K27-SAD2g-R	GTCTCGTCGGTTGTTTACTGG
h3k27-sad9g-F	CCTACTCGTGCCAGCCCTATC
h3k27-sad9g-R	CGCTCGTCGGTGAACCAATCT
h3k27-sad7g-F	ACGACGCCTTCTTCTTCTTCTTC
h3k27-sad7g-R	TGTCCGACTCCTCACACTACTCAG
h3k27-sad10g-F	GCGAAACCTATCGAACCTCTGC
h3k27-sad10g-R	CCCATTCCCATCTTGACGTGT

References

59. Hu, Y. F., Liu D., Zhong X., Zhang C., Zhang Q., Zhou D.X. (2012) CHD3 protein recognizes and regulates methylated histone H3 lysines 4 and 27 over a subset of targets in the rice genome. *Proc. Natl. Acad. Sci. U.S.A.*, 109, 5773-5778.

60. Sonderby I. E., Geu-Flores F., Halkier B. A. (2010) Biosynthesis of glucosinolates - gene discovery and beyond. *Trends Plant Sci.* **15**, 283-290.

61. Saito K. *et al.* (2013) The flavonoid biosynthetic pathway in *Arabidopsis*: structural and genetic diversity. *Plant. Physiol. Biochem.* **72**, 21-34.

62. Hruz T., Laule O., Szabo G., Wessendorp F., Bleuler S., Oertle L., Widmayer P., Gruissem W., Zimmermann P. (2008) Genevestigator V3: a reference expression database for the meta-analysis of transcriptomes. *Advances in Bioinformatics* **2008**, 420747.

Supplementary script: Python script for cluster mining

```
#!/usr/bin/python

import networkx as nx
import matplotlib.pyplot as plt
from networkx.utils import is_list_of_ints, flatten
from operator import itemgetter
import matplotlib.patches as pat
import math, getopt, sys, numpy, random
from collections import OrderedDict

# a tab separated file containing columns: gene1 gene2 correlation
# where correlations were only calculated between genes on the same
chromosome
corr_file = "maize_corr_mat_chr_all.dat"

# a file containing an list of genes ordered by location
gene_order_file = "sort_gene_list_chr_all_no_dupes.dat"

# a tab separated file containing columns: gene1 gene2
# where the two genes where found to be homologues of each other using
BLASTn
dupe_file = "./blast/duplicate_pairs.dat"

letters = 'c:o:d:'
opts, params = getopt.getopt(sys.argv[1:], letters)
for o, p in opts:
    if o == "-c":
        corr_file = p
    if o == "-o":
        gene_order_file = p
    if o == "-d":
        dupe_file = p


gL = open(gene_order_file)
lc = 0
gene_index={}
for line in gL:
    g_cols = line.split('\t')
    item = g_cols[0]
    item = item.rstrip()
    # check for duplicates
    if item not in gene_index:
        gene_index[item.rstrip()] = lc
        lc+=1




G = nx.Graph()
count = 0
dupe = []
dupec = 0
errc = 0
```

```
tot = 0
dupes = open(dupe_file, "r")
for line in dupes:
    cols = line.split('\t')
    key = cols[0].rstrip() + cols[1].rstrip()
    dupe.append(key)



corr_h = open(corr_file, "r")
for val in corr_h:
    if not val.startswith('\"\",\"'):
        cols=val.split('\t')
        tot +=1
        g1 = cols[0]
        g2 = cols[1]
      corr = float(cols[2])
      ids = g1+g2
        if g1 in gene_index and g2 in gene_index:
            gene_dist = abs(gene_index[g1] - gene_index[g2])
        else:
            gene_dist = 9999999
        if float(cols[2]) > 0.65:
            if (g1+g2) not in dupe and (g2+g1) not in dupe:
                try:
                    # Set graph structure
                    if gene_dist < 10:
                        count +=1
                        if g1 not in G.nodes():
                            G.add_node(g1)
                        if g2 not in G.nodes():
                            G.add_node(g2)
                        G.add_edge(g1, g2, weight=corr)
                except:
                    errc +=1
            else:
                dupec +=1




# remove all two gene clusters
for edge in G.edges():
    if G.degree(edge[0]) == 1 and G.degree(edge[1]) == 1:
        G.remove_nodes_from(edge)

outConnComp = open(gene_order_file + "_SUB.csv","w")
outCliques = open(gene_order_file + "_CLIQ.csv","w")


# find all subgraphs
cl = nx.connected_components(G)
sub_num = 1
for c in cl:
    outConnComp.write("sub:"+str(sub_num)+"\t")
    sub_num += 1
    for i_ in sorted(c):
```

```
        outConnComp.write(i_ + "\t")
    outConnComp.write("\n")




# find all cliques
nl = list(nx.find_cliques(G))
connodes = list(set([n for sub in nl for n in sub if len(sub) > 2]))
for node in G.nodes():
    if node not in connodes:
        G.remove_node(node)
cl2 = nx.connected_components(G)
cliq_num=1
for c2 in cl2:
    outCliques.write("cliq:"+str(cliq_num)+"\t" )
    cliq_num += 1
    for i2 in sorted(c2):
        outCliques.write( i2 + "\t")
    outCliques.write("\n")
```
